# OTUD4 enhances TGFβ signalling through regulation of the TGFβ receptor complex

**DOI:** 10.1038/s41598-020-72791-0

**Published:** 2020-09-24

**Authors:** Patrick William Jaynes, Prasanna Vasudevan Iyengar, Sarah Kit Leng Lui, Tuan Zea Tan, Natali Vasilevski, Sarah Christine Elizabeth Wright, Giuseppe Verdile, Anand D. Jeyasekharan, Pieter Johan Adam Eichhorn

**Affiliations:** 1 Cancer Science Institute of Singapore, National University of Singapore, Singapore, 117599 Singapore; 2 Department of Cell and Chemical Biology and Oncode Institute, Leiden University Medical Center, 2333 ZC Leiden, The Netherlands; 3 School of Pharmacy and Biomedical Sciences, Faculty of Health Sciences, Curtin University, Bentley, 6102 Australia; 4 Curtin Health Innovation Research Institute and Faculty of Health Sciences, Curtin University, Bentley, WA 6102 Australia; 5 School of Medical and Health Sciences, Edith Cowan University, Joondalup, WA 6027 Australia; 6 Department of Pharmacology, Yong Loo Lin School of Medicine, National University of Singapore, Singapore, 117597 Singapore

**Keywords:** Deubiquitylating enzymes, Ubiquitylation, Transforming growth factor beta, Cancer

## Abstract

Systematic control of the transforming growth factor-β (TGFβ) pathway is essential to keep the amplitude and the intensity of downstream signalling at appropriate levels. Ubiquitination plays a crucial role in the general regulation of this pathway. Here we identify the deubiquitinating enzyme OTUD4 as a transcriptional target of the TGFβ pathway that functions through a positive feedback loop to enhance overall TGFβ activity. Interestingly we demonstrate that OTUD4 functions through both catalytically dependent and independent mechanisms to regulate TGFβ activity. Specifically, we find that OTUD4 enhances TGFβ signalling by promoting the membrane presence of TGFβ receptor I. Furthermore, we demonstrate that OTUD4 inactivates the TGFβ negative regulator SMURF2 suggesting that OTUD4 regulates multiple nodes of the TGFβ pathway to enhance TGFβ activity.

## Introduction

The Transforming Growth Factor β (TGFβ) pathway is crucial for embryonic development as well as maintaining tissue homeostasis in adult tissues. The majority of human cell types are responsive to TGFβ, by which it can alter cellular differentiation, cell proliferation, migration, adhesion, and immune surveillance^1–3^. Activation of the TGFβ pathway requires the ligand induced formation of a tetrameric complex comprised of two TGFβ receptor I (TβRI) subunits as well as two TGFβ receptor II (TβRII) subunits^4^. TGFβ receptor (TβR) complex formation results in the activation of TβRI. Activated receptors induce intracellular signalling through the phosphorylation of receptor-regulated SMADs (R-SMADs), specifically SMAD2 and SMAD3, in the TGFβ pathway^1,3^. Once phosphorylated R-SMADs associate with the co-SMAD, SMAD4, and upon entry into the nucleus the R-SMAD/co-SMAD complex binds to conserved SMAD binding element (SBE) sequences, driving transcription^2,3,5^. To ensure that external cellular cues generate desired intracellular responses, inhibitory feedback loops exist to limit unwanted prolonged hyperactivation of the pathway.

In the case of TβR signalling, SMAD7 and USP26, direct transcriptional targets of the SMAD complexes, function through a negative feedback loop to attenuate TGFβ signalling^6–9^. USP26 deubiquitinates and stabilizes SMAD7, permitting SMAD7 to act as scaffold to recruit the E3 ligase SMURF2 to the TβR complex thereby facilitating ubiquitin mediated proteasomal degradation of the receptor complex^6–9^. Besides acting as a scaffold, SMAD7 can also act as an agonist for SMURF2 activity on a number of different levels. SMURF2 contains a C2 domain, three WW domains and a C-terminal HECT domain^10^. To limit unnecessary activity towards its substrates, the N-terminal C2 domain interacts with the C-terminal HECT domain inhibiting ubiquitin thioester bond formation of its catalytic cysteine residue. The binding of SMAD7 to the WW3-HECT domain of SMURF2 overcomes the inhibitory intramolecular interactions between these domains, opening up SMURF2 and facilitating SMURF2 ubiquitin ligase activity^11,12^. Furthermore, SMAD7 permits the association of SMURF2 with the E2 ligase UBCH7^9^.

Ubiquitination is an ATP dependent process by which ubiquitin, a 76 amino acid protein, is conjugated to lysine residues on a protein substrate^13^. This process involves the coordinated activity of 3 enzymes simply designated E1, E2 and E3^14^. Polyubiquitin chains are strings of ubiquitin moieties attached via isopeptide bonds that are formed between the carboxyl terminus of a distal ubiquitin and the ε-amino group of a given lysine in the preceding (proximal) ubiquitin in the chain^15^. Polyubiquitin chains can utilize lysine 6 (K6), lysine 11 (K11), lysine 27 (K27), lysine 29 (K29), lysine 33 (K33), lysine 48 (K48) and lysine 63 (K63) as well as the N-terminal Met1 residue for their isopeptide linkages^13^. The physiological relevance of polyubiquitin conjugates is varied, with the effects of K48 and K63 chain types being the most well studied^13^. K48 chains are the most abundant across all organisms and are known to target proteins for degradation in the 26S proteasome^15^. K63 chains, in contrast, have been found to be involved in non-proteolytic processes such as protein kinase activation^16^.

Ubiquitin plays a crucial role in regulating endocytosis, acting as an internalization signal for the endocytic machinery^17^. The TβR complex has two distinct routes of endocytosis; either into clathrin coated pits or into lipid-raft caveolin positive vesicles^18^. SMURF2 and SMAD7 only interact with and promote degradation of the TβR complex in the caveolin positive compartment^18^. Indeed, there is already an abundance of evidence demonstrating that pro-degradative K48-ubiquitin linked chains are crucial for regulation of the TGFβ signalling^8^. However, a paucity of information is available regarding ubiquitin’s role in the endocytosis of receptors of this pathway and the agents which regulate ubiquitin chain topologies. Here we identify the deubiquitinating enzyme OTUD4 as a positive regulator of TGFβ signalling, functioning through a positive feedback loop to regulate TβR activation. Importantly, it is clear from our data that OTUD4 does not only regulate K48-linked ubiquitin chains required for proteasomal degradation but rather atypical chain topologies that appear to be important for the plasma membrane presence and endocytic sorting of TβR.

## Results

### OTUD4 activates the canonical TGFβ pathway

**Figure 1:**
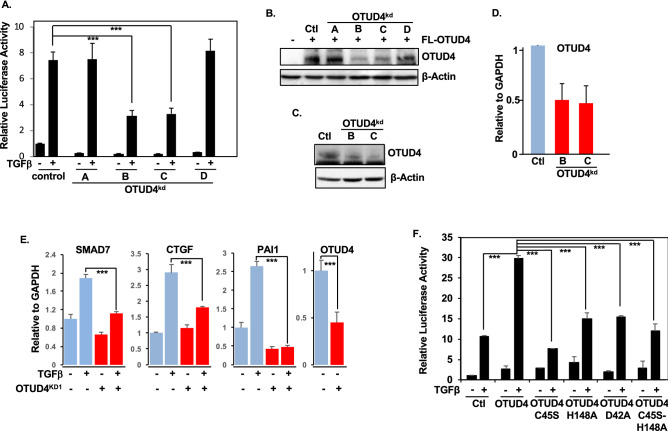
OTUD4 activates the canonical TGFβ pathway. (**A**) TGFβ responsive luciferase (CAGA luciferase) of HEK293T cells transfected with four independent OTUD4 shRNA hairpins labelled A, B, C and D. Cells were stimulated where indicated with TGFβ (100 pM) overnight before lysis. Error bars represent SD of triplicates. Experiment is a representative of 3 independent experiments. ***P ≤ 0.001 as determined by Student’s T-Test. (**B**) Western blot analysis of HEK23T cells transfected with FLAG-OTUD4 and OTUD4 knockdown shRNA hairpins A, B, C and D. β-Actin is used as the loading control. (**C**) Western blot analysis of HEK293T cells transfected with OTUD4 knockdown shRNA hairpins B and C. Immunoblotting for OTUD4 was performed. β-Actin is used as the loading control. (**D**) HEK293T cells were transfected with OTUD4 knockdown constructs B and C or relevant controls. *OTUD4* mRNA levels relative to GAPDH are shown as evaluated by quantitative real-time PCR. Data are shown as the mean ± SD of triplicate samples from a representative experiment performed three times. (**E**). HEK293T OTUD4^KD1^ cells were stimulated with TGFβ (100 pM) for 3 h. *SMAD7, CTGF, PAI1 and OTUD4* mRNA levels relative to GAPDH are shown as evaluated by quantitative real-time PCR. Data are shown as the mean ± SD of triplicate samples from a representative experiment performed three times. ***P ≤ 0.001 as determined by Student’s T-Test. (**F**) TGFβ responsive luciferase (CAGA luciferase) of HEK293T cells transfected with FLAG-OTUD4 WT, C45S, H148A, D42A, or C45S-H148A. Cells were stimulated where indicated with TGFβ (100 pM) overnight before lysis. Data are shown as the mean ± SD of triplicate samples from a representative experiment performed three times. ***P ≤ 0.001 as determined by Student’s T-Test. Full-length blots for (**B**,**C**) are shown in Supplementary Information.

Previously, we performed an shRNA deubiquitinating enzyme screen to uncover novel deubiquitinating enzymes (DUBs) in the TGFβ pathway. From this we identified OTUD4 as a potential activator of TGFβ activity^19^. This shRNA library consists of 100 pools of four non-overlapping shRNAs targeting all known or putative DUBs^20^. To validate our results from our original screen we first isolated the four shRNA hairpins from the OTUD4 DUB pool and tested which of the OTUD4 shRNA hairpins could inhibit the activity of a TGFβ responsive luciferase reporter (CAGA-luc). shRNA B and C significantly inhibited luciferase activity compared to a control hairpin targeting GFP (Fig. 1A). Next, we tested whether hairpins B and C could effectively inhibit OTUD4 protein expression. As expected, shRNA B and C inhibited both ectopically expressed and endogenous OTUD4 levels (Fig. 1B,C). Moreover, these hairpins effectively inhibited OTUD4 expression as determined by quantitative reverse transcriptase PCR (qRT-PCR) (Fig. 1D). To further confirm the role of OTUD4 in regulating the canonical TGFβ pathway we analyzed the expression of TGFβ target genes in cell lines stably expressing shRNA vectors targeting OTUD4 (Sup. Fig. 1A). Consistent with our observations thus far, depletion of OTUD4 downregulated *SMAD7, CTGF*, and *PAI1* mRNA levels (Fig. 1E).

Given that OTUD4 depletion diminishes TGFβ signalling, we next examined the effect of ectopic OTUD4 expression on TGFβ activity. Utilizing the previously mentioned CAGA-luc reporter system, we found that overexpression of wild type OTUD4, in either the presence or absence of TGFβ ligand, resulted in enhanced luciferase activity (Fig. 1F). We next proceeded to investigate the importance of the OTUD4’s deubiquitinase activity for this augmentation. OTUD4 is a cysteine based isopeptidase utilizing a catalytic cysteine at the 45 amino acid position and a histidine at position 148 for its nucleophilic attack^16,21^. Along with these residues, an aspartic acid at position 42, completing the catalytic triad, is speculated to be required for full activation of OTUD4 deubiquitinase activity^16,21^ (Sup. Fig. 1B). As shown in Fig. 1F, the TGFβ induced luciferase activity was severely mitigated upon ectopic expression of each of the three catalytic OTUD4 mutants C45S, H148A, D42A as well as the dual mutant, C45S-H148A (henceforth referred to as Dub Dead (DD)). Taken together these results indicate that OTUD4 is regulator of TGFβ activity and that this regulation is, at least in part, dependent on OTUD4’s catalytic activity.

### OTUD4 regulates SMAD phosphorylation

**Figure 2:**
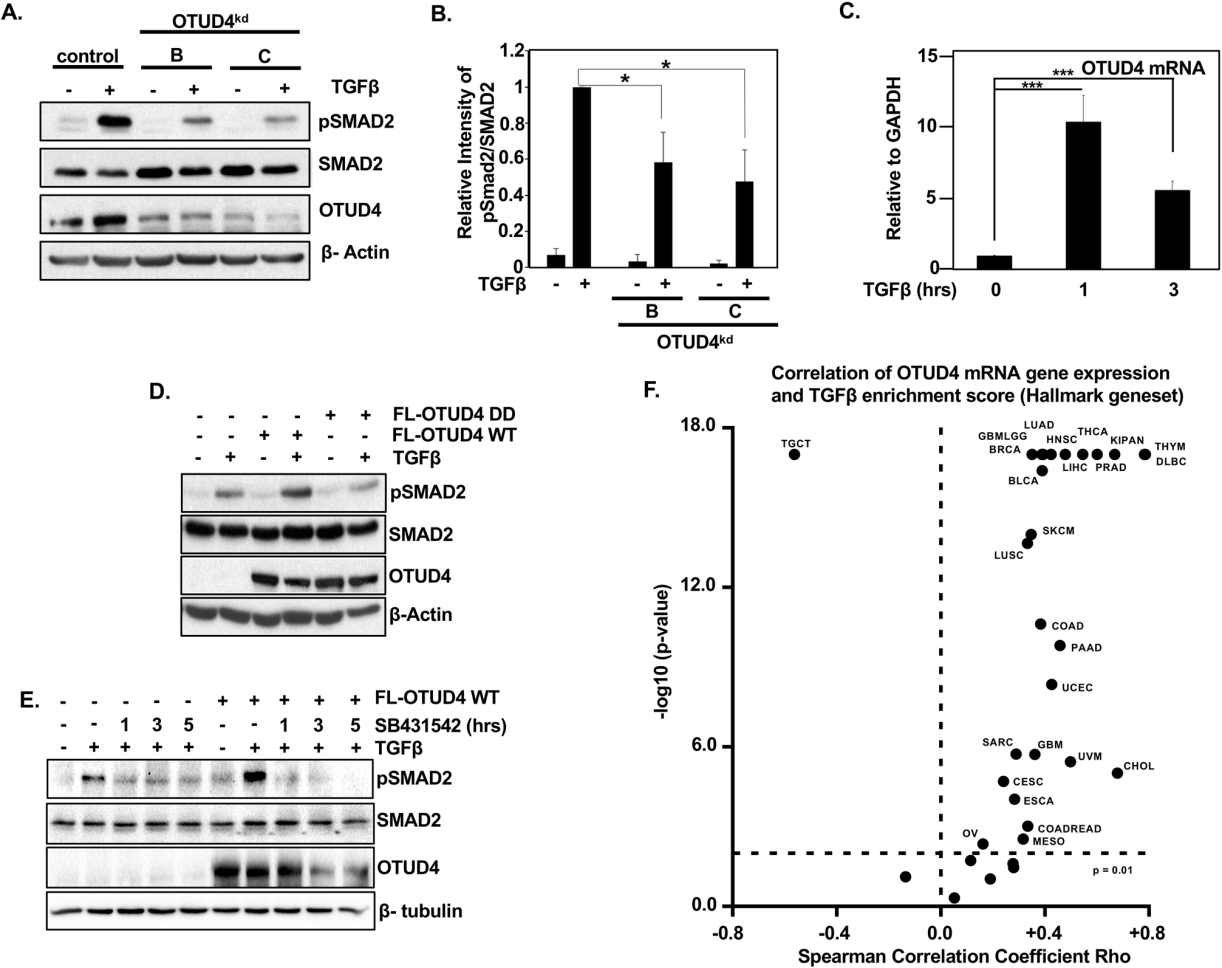
OTUD4 regulates TGFβ receptor activity. (**A**) HEK293T cells transfected with OTUD4 shRNA hairpins B and C. Cells were stimulated where indicated with TGFβ (100 pM) overnight before lysis. Whole cell extracts were probed with indicated antibodies. β-Actin is used as the loading control. (**B**) Quantification of pSMAD2 levels represented in (**A**). Data are shown as the mean ± SD of 3 independent experiments. *P < 0.05 as determined by Student’s T-Test. (**C**) HEK293T stimulated with TGFβ (100 pM) for 1 or 3 h. *OTUD4* mRNA levels relative to GAPDH are shown as evaluated by quantitative real-time PCR. Data are shown as the mean ± SD of triplicate samples from a representative experiment performed three times. ***P ≤ 0.001 as determined by Student’s T-Test. (**D**) HEK293T cells transfected with FLAG-OTUD4, or FLAG OTUD4 DD (C45S-H148A). Cells were stimulated where indicated with TGFβ (100 pM) overnight before lysis. Whole cell extracts were probed with indicated antibodies. β-Actin is used as the loading control. (**E**) HEK293T cells transfected with FLAG-OTUD4 were stimulated where indicated with TGFβ (100 pM) for 1 h prior to SB431542 (1 µM). Cells were collected as indicated and whole cell extracts were probed with indicated antibodies. β-Tubulin is used as the loading control. (**F**) Spearman’s analysis between OTUD4 mRNA expression and TGFβ enrichment score (Hallmark geneset) score across a TCGA pan-cancer dataset (n = 12,290). Full-length blots for (**A**,**D**,**E**) are shown in Supplementary Information.

As OTUD4 is required for TGFβ induced transcriptional responses, we investigated the role of OTUD4 on TGFβ intercellular signalling in detail. To this end we compared the levels of phosphorylated SMAD2 (pSMAD2), which acts as a proxy for TGFβ receptor activity, in HEK293T cells transfected with shRNAs targeting OTUD4 or relevant controls. As expected, TGFβ ligand enhanced SMAD2 phosphorylation levels in control conditions. In contrast, depletion of OTUD4 significantly decreased pSMAD2 levels while having no effect on the overall levels of SMAD2 (Fig. 2A,B). Similar effects were observed in HEK293T cells stably expressing shRNA hairpins targeting OTUD4 (Sup. Fig. 1C). Interestingly, we noted that upon the addition of TGFβ, OTUD4 protein levels significantly increased indicating that OTUD4 expression may be directly controlled by TGFβ signalling (Fig. 2A, Sup. Fig. 1C). To determine whether the changes on OTUD4 levels were dependent on TGFβ mediated transcription we analysed *OTUD4* mRNA levels following TGFβ exposure. Indeed, *OTUD4* mRNA levels were increased following the addition of TGFβ ligand suggesting that *OTUD4* is a transcriptional target of the canonical TGFβ pathway (Fig. 2C).

Since knockdown of OTUD4 inhibited phosphorylated SMAD2, we next asked if ectopic expression of OTUD4 enhanced pSMAD2. Consistent with our knockdown results, ectopic expression of OTUD4 wild type, but not OTUD4 DD, increased the levels of p-SMAD2 (Fig. 2D). To further confirm whether OTUD4-mediated pSMAD2 regulation was dependent upon canonical TGF-β receptor signalling, we ectopically expressed OTUD4 and treated the cells with TGF-β ligand for 1 h prior to adding TβRI inhibitor SB431542, and analysed pSMAD2 levels over time^22,23^. As previously observed, OTUD4 robustly upregulated SMAD2 phosphorylation levels, however, in the presence of SB431542, SMAD2 phosphorylation was completely annulled at all the time points analysed (Fig. 2E). As SB4341542 precluded OTUD4’s ability to influence downstream TGFβ signalling it strengthens the notion that OTUD4 operates at the TGFβ receptor level to regulate pathway signalling. Taken together, these results suggest that OTUD4 regulates TGFβ signalling upstream of the SMAD2/3 transcription factor complex, possibly at the receptor level.

Next, we sought to determine if OTUD4 expression correlated with TGFβ signalling in cancer. To this end we probed the TCGA pan-cancer dataset (n = 12,290) and correlated OTUD4 expression with TGFβ pathway activation using the MSigdb v6.1 Hallmark TGFβ signature. We found that OTUD4 expression significantly correlated (Spearman correlation, p < 0.01) with the Hallmark TGFβ signature enrichment score in 25 of the 37 tumour types tested (Fig. 2F, Sup. Table 1). Interestingly, in all but one of these tumour types OTUD4 expression positively correlated with TGFβ activation, whilst only in testicular germ cell tumours (TGCT) was OTUD4 expression negatively correlated with TGFβ activity. This suggests that OTUD4 expression corresponds with TGFβ signalling in the majority of tumour types including breast, glioblastoma, and diffuse large B cell lymphoma and may regulate TGFβ signalling in these cancers. To further test whether OTUD4 regulates the TGFβ pathway in breast cancer we knocked down OTUD4 in the breast cancer cell lines MCF7 and MDA-MB-231. In line with our previous results, depletion of OTUD4 decreased overall pSMAD2 levels compared to controls following TGFβ ligand addition. (Sup Fig. 1D,E). Taken together these results suggests that OTUD4 is an integral regulator of the TGFβ pathway in multiple tumour types.

### OTUD4 regulates the ubiquitination of the TβR complex

**Figure 3:**
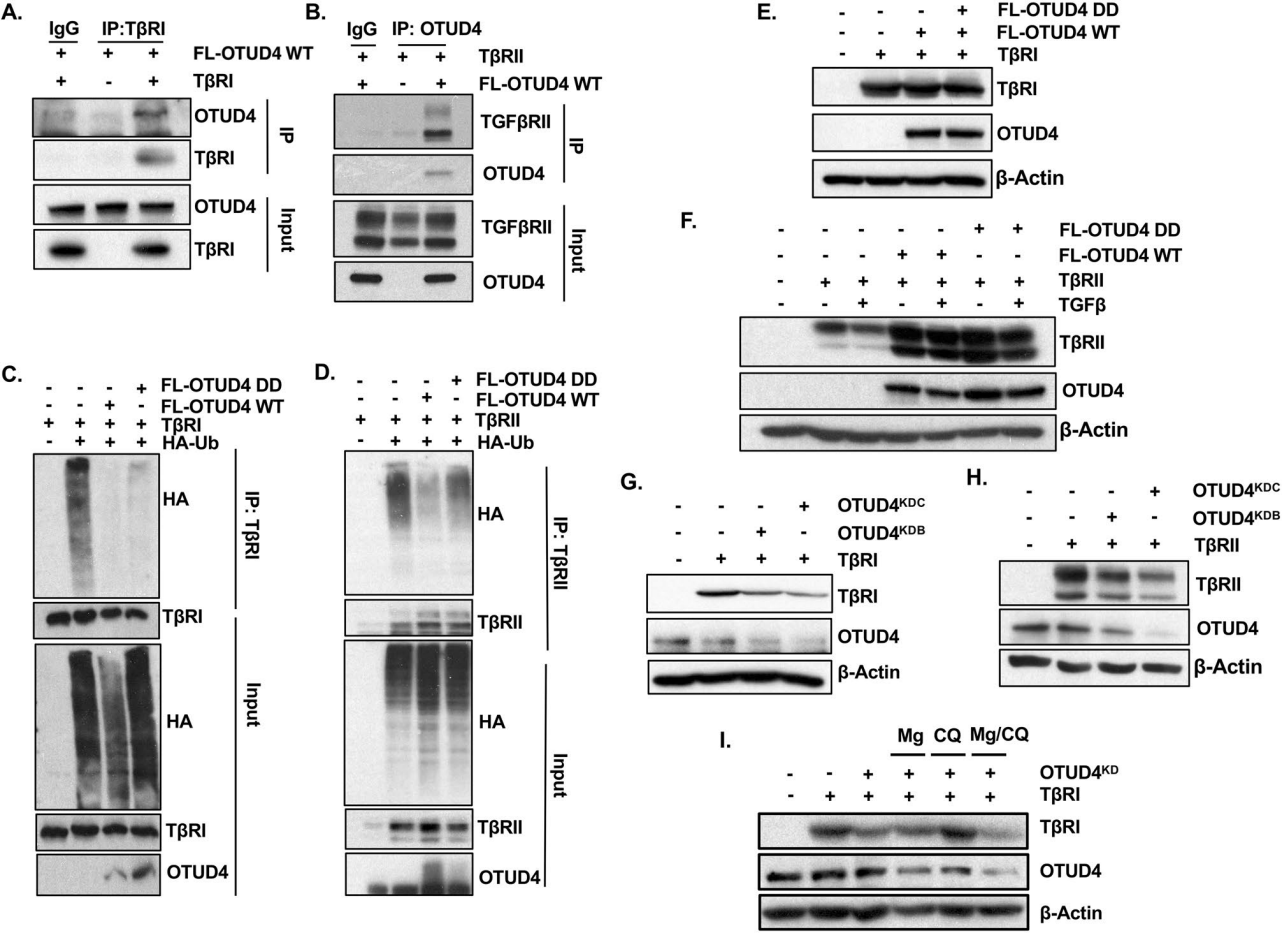
OTUD4 regulates the ubiquitination of the TβR complex. (**A**) HEK293T cells were transfected with TβRI and/or FLAG-OTUD4. After 48 h cells were lysed and immunoprecipitated with anti- TβRI affinity resin. Immunoprecipitated lysates and whole cell extracts were probed with the indicated antibodies. (**B**) HEK293T cells were transfected with TβRII and FLAG-OTUD4. After 48 h cells were lysed and immunoprecipitated with anti- OTUD4 affinity resin. Immunoprecipitated lysates and whole cell extracts were probed with the indicated antibodies. (**C**) HEK293T cells were transfected with HA-ubiquitin, TβRI and either FLAG-OTUD4, or FLAG-OTUD4 DD. After 48 h cells were lysed and immunoprecipitated with anti- TβRI affinity resin. Immunoprecipitated lysates and whole cell extracts were probed with the indicated antibodies. (**D**) HEK293T cells were transfected with HA-ubiquitin, TβRII and either FLAG-OTUD4, or FLAG-OTUD4 DD. After 48 h cells were lysed and immunoprecipitated with anti-TβRII affinity resin. Immunoprecipitated lysates and whole cell extracts were probed with the indicated antibodies. (**E**) HEK293T cells were transfected with TβRI and either FLAG-OTUD4 or FLAG OTUD4 DD. Whole cell extracts were probed with indicated antibodies. β-Actin is used as the loading control. (**F**) HEK293T cells were transfected with TβRII and either FLAG-OTUD4 or FLAG OTUD4 DD. Cells were stimulated where indicated with TGFβ (100 pM) overnight before lysis. Whole cell extracts were probed with indicated antibodies. β-Actin is used as the loading control. (**G**) HEK293T cells were co-transfected with TβRI and OTUD4 shRNA hairpins B and C. After 72 h cells were lysed and whole cell extracts were probed with indicated antibodies. β-Actin is used as the loading control. (**H**) HEK293T cells were co-transfected with TβRII and OTUD4 shRNA hairpins B and C. After 72 h cells were lysed and whole cell extracts were probed with indicated antibodies. β-Actin is used as the loading control. (**I**) HEK293T cells were co-transfected with TβRI and OTUD4 shRNA hairpin C. Cells were treated with either MG132 (10 μM) or chloroquine (400 μM) or in combination overnight before lysis. Cells were subsequently lysed and whole cell extracts were probed with indicated antibodies. β-Actin is used as the loading control. Full-length blots for all panels are shown in Supplementary Information.

Previously, it has been demonstrated that a number of DUBs control overall TGFβ activity through regulation of TGFβ receptor dynamics^19,24–27^. We therefore sought to validate whether or not OTUD4 interacted with components of the TGFβ receptor complex. Indeed, immunoprecipitation involving either the TβRI or TβRII subunit confirmed an association with OTUD4 (Fig. 3A,B). As OTUD4 formed a complex with TβR, we sought to determine if TβR was a direct target of OTUD4′s deubiquitinase activity. To this end, we ectopically expressed either TβRI or TβRII and HA-tagged ubiquitin with OTUD4 or OTUD4 DD. TβRI or TβRII were affinity purified and their ubiquitination patterns verified with an antibody targeting HA. We observed that OTUD4 decreased the levels of incorporated ubiquitin on both TβRI and TβRII (Fig. 3C,D). However, contrary to expectations, OTUD4 DD also decreased the overall levels of TβRI ubiquitination while only partially rescuing TβRII ubiquitination levels. Neither ectopic expression of OTUD4 WT nor OTUD4 DD enhanced overall TβRI levels with respect to controls, suggesting that OTUD4 may not regulate the cleavage of pro-degradative K48-ubiquitin linked chains in this context (Fig. 3E). Interestingly, OTUD4 did stabilize TβRII levels but similar results were also observed with OTUD4 DD, suggesting that this stabilization was not a direct result of OTUD4’s deubiquitinase activity (Fig. 3F). We then sought to address whether OTUD4 knockdown altered the expression levels of the TβR complex. Cells depleted for OTUD4 displayed decreased levels of TβRI and TβRII (Fig. 3G,H).

Taken together, our results thus far suggest that OTUD4-mediated deubiquitination of the TβR complex is unlikely to solely regulate K48 pro-degradative ubiquitin topologies. Consistent with this, treatment of OTUD4 depleted cells with the proteasome inhibitor MG132 did not rescue TβRI destabilization (Fig. 3I). Interestingly, however, in this system, treatment with the lysosomal inhibitor, chloroquine, annulled the downregulation of TβRI by OTUD4 knockdown. Collectively, these results demonstrate that OTUD4 regulates the ubiquitination of the TβR complex through both catalytic and catalytic independent functions and may partly function to modify endosomal sorting of the TβR complex.

### OTUD4 regulates TβRI presence at the plasma membrane

**Figure 4:**
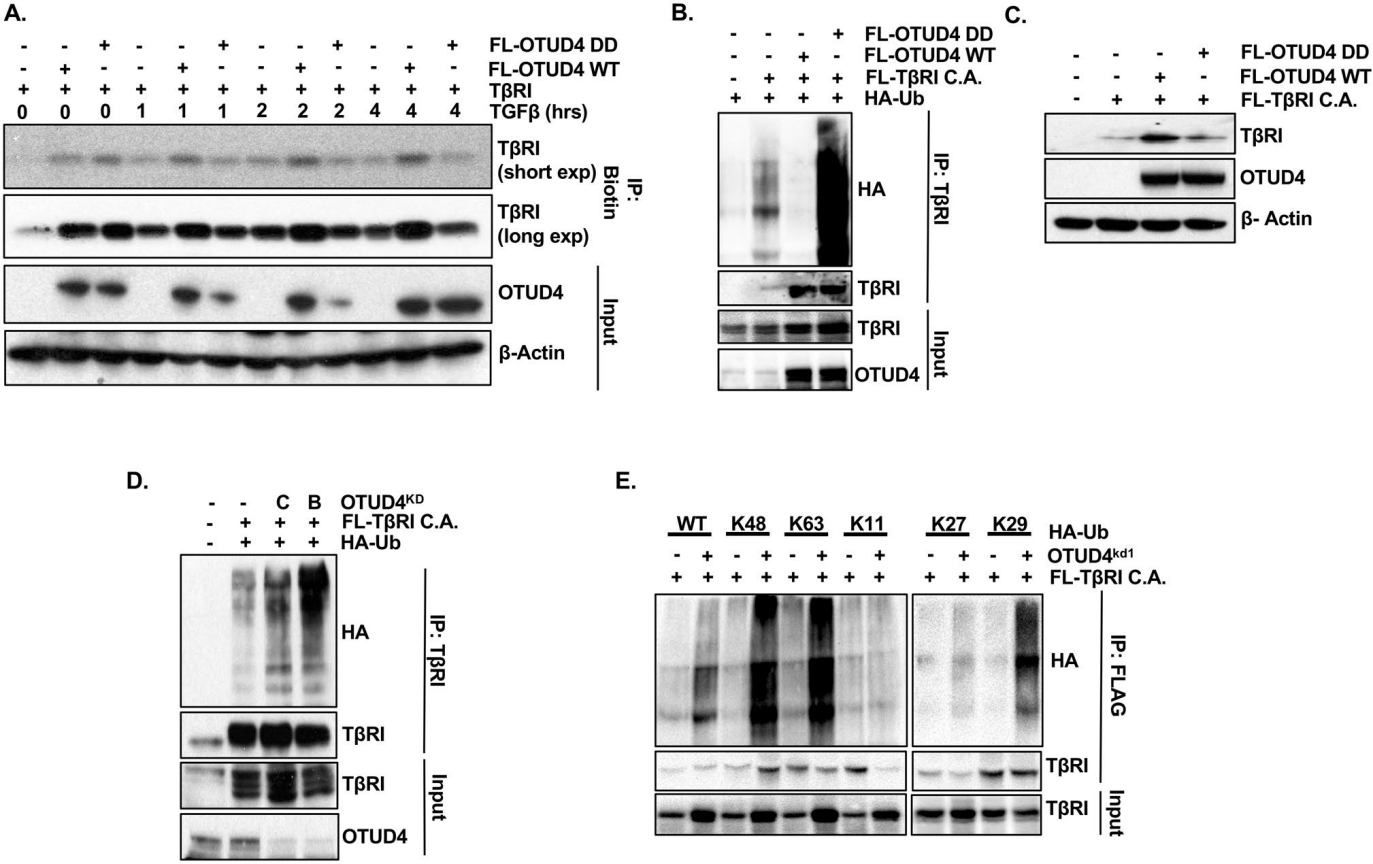
OTUD4 regulates TβRI presence at the plasma membrane. (**A**) HEK293T cells were transfected with TβRI and either FLAG-OTUD4 or FLAG-OTUD4 DD. Cells were stimulated with TGFβ (100 pM) for the length of time indicated. Cell surface was labelled with biotin at 4 degrees for 40 min before lysis. Lysates were immunoprecipitated with anti- NeutrAvidin affinity resin. Immunoprecipitated lysates and whole cell extracts were probed with the indicated antibodies. β-Actin is used as the loading control. (**B**) HEK293T cells were transfected with HA-ubiquitin, FLAG-TβRI C.A. and either FLAG-OTUD4 or FLAG-OTUD4 DD. Cells were incubated with MG132 (5 µM) overnight before lysis. After 48 h cells were lysed and immunoprecipitated with anti-TβRI affinity resin. Immunoprecipitated lysates and whole cell extracts were probed with the indicated antibodies. (**C**) HEK293T cells were transfected with FLAG-TβRI C.A. and either FLAG-OTUD4 or FLAG OTUD4 DD. Whole cell extracts were probed with indicated antibodies. β-Actin is used as the loading control. (**D**) HEK293T cells were transfected with HA-ubiquitin, FLAG-TβRI C.A. and OTUD4 shRNA constructs B or C. Cells were incubated with MG132 (5 µM) overnight before lysis. After 72 h cells were lysed and immunoprecipitated with anti-TβRI affinity resin. Immunoprecipitated lysates and whole cell extracts were probed with the indicated antibodies. (**E**) HEK293T OTUD4^KD1^ cells were transfected with FLAG-TβRI C.A. and either HA-ubiquitin (WT), HA-K48 ubiquitin, HA-K63 ubiquitin, HA-K11 ubiquitin, HA-K27 ubiquitin or HA-K29 ubiquitin. Cells were incubated with MG132 (5 µM) overnight before lysis. Immunoprecipitated lysates and whole cell extracts were probed with the indicated antibodies. Full-length blots for all panels are shown in Supplementary Information.

Receptor ubiquitination has also been reported to promote their internalization and subsequent trafficking to the lysosomes, resulting in enhanced pathway activation or receptor recycling^28–31^. It is therefore possible that OTUD4 may be an important factor in the endocytosis and/or trafficking of TβR complex. With this in mind we turned our attention to OTUD4’s potential effect on the endocytosis of the TβR complex. For this investigation we focused on TβRI as this subunit directly phosphorylates downstream effector proteins and is therefore the rate-limiting factor for pathway activation. In order to measure how OTUD4 affects endocytosis we labelled TβRI expressing HEK293T cells with biotin and performed a biotin pulldown to assess how either OTUD4 or OTUD4 DD might affect the cell surface expression of this subunit. Interestingly, in the absence of TGFβ, both OTUD4 and OTUD4 DD increased TβRI levels at the plasma membrane (Fig. 4A). Importantly, however, following exposure to TGFβ ligand, OTUD4 but not OTUD4 DD enhanced cell surface TβRI levels (Fig. 4A).

We next wanted to verify this phenomenon at the single cell level via confocal immunofluorescence microscopy. Similar to the biotin experiment we found that in the presence of TGFβ ligand TβRI membrane localization was maintained in cells ectopically expressing OTUD4 but to a lesser degree in cells expressing the catalytically inactive version (Fig. Sup. 2). However, as we did not see this phenomenon in every cell examined, we conclude that OTUD4 indeed increases the likelihood of TβRI localization at the membrane but that this phenomenon is best evaluated at the population level. Overall, this data alludes to the notion that in the presence of the ligand, which promotes receptor dimerization and activation, OTUD4-mediated deubiquitination is required to maintain TβR complex at the plasma membrane perhaps by inhibiting endocytosis.

To further ascertain whether the ligand-dependent differences between OTUD4 and OTUD4 DD activity is due to TβRI ubiquitination, we investigated how OTUD4 affected the ubiquitin profile of activated TβRI. To mimic ligand-induced receptor activation we made use of a constitutively active version of TβRI: TβRI C.A. In agreement with our previous result (Fig. 3C), OTUD4 decreased the levels of ubiquitinated TβRI C.A. (Fig. 4B). However, in this context, OTUD4 DD significantly increased the levels of TβRI C.A. ubiquitination, unlike what we observed with wild type TβRI. This implies the catalytic activity of OTUD4 is required to remove ubiquitin chains specifically associated with TβRI activation. This suggests that the phenomenon observed in Fig. 4A where, in the presence of ligand, TβRI membrane localization is diminished in cells expressing OTUD4 DD, is a direct result of ubiquitination.

Interestingly, overexpression of OTUD4, but not OTUD4 DD, markedly increased the expression of TβRI C.A. (Fig. 4C). This phenomenon was not seen with wild type TβRI (Fig. 3E) and may possibly reflect OTUD4′s ability to perturb the trafficking of activated TβRI to the lysosome following endocytosis. Next, we sought to address if OTUD4 knockdown increases the levels of TβRI C.A. ubiquitination. As observed in Fig. 4D, both OTUD4 specific shRNAs, in the presence of the proteasome inhibitor MG132, significantly enhanced the levels of ubiquitinated TβRI. We next wanted to identify the nature of these OTUD4-regulated polyubiquitin chains. We therefore analyzed whether loss of OTUD4 influenced the levels of TβRI K11, K27, K29, K48, and K63 ubiquitination. HEK293T cells stably expressing OTUD4 knockdown vector 1 (KD1) were transfected with FLAG-tagged TβRI C.A. and either wild type Ub or respective K11/K27/K29/K48/K63 Ub variants. In these mutants all the lysine residues have been replaced with arginine residues except at the denoted residue, which remains a lysine. Among these mutants, depletion of OTUD4 significantly enhanced K48, K63, and K29 ubiquitin chains while having no effect on K11 or K27 ubiquitin chains (Fig. 4E). Therefore, we speculate that OTUD4 adjusts TβRI localization and expression through multiple modes of ubiquitin-mediated regulation marked by variations in K29, K48, and K63 chain topologies. This may then shunt TβRI through different endosomal compartments either enhancing signalling or targeting the receptor for proteasome-independent degradation. However, these results do not address the non-catalytic functions of OTUD4.

### OTUD4 regulates SMURF2 auto-regulation

That the deubiquitination of TβRI, in the absence of ligand-induced activation, is independent of OTUD4’s catalytic activity, implies that OTUD4 may subvert the action of other components of the TGFβ receptor complex. We therefore turned our attention to proteins that are known to modify the ubiquitin status of the TβR complex. As previously discussed, the E3 ligase SMURF2 resides in a closed confirmation with the C2 domain of the protein coming in close contact with the catalytic HECT domain. As SMURF2 can undergo autoubiquitination, this inhibitory confirmation regulates both SMURF2 stability and unwanted ubiquitination of non-specific target substrates. The binding of SMAD7 abrogates these intramolecular interactions permitting SMURF2 autoubiquitination and ligase activity towards its substrates including the TGFβ receptor complex^6,12^.

**Figure 5:**
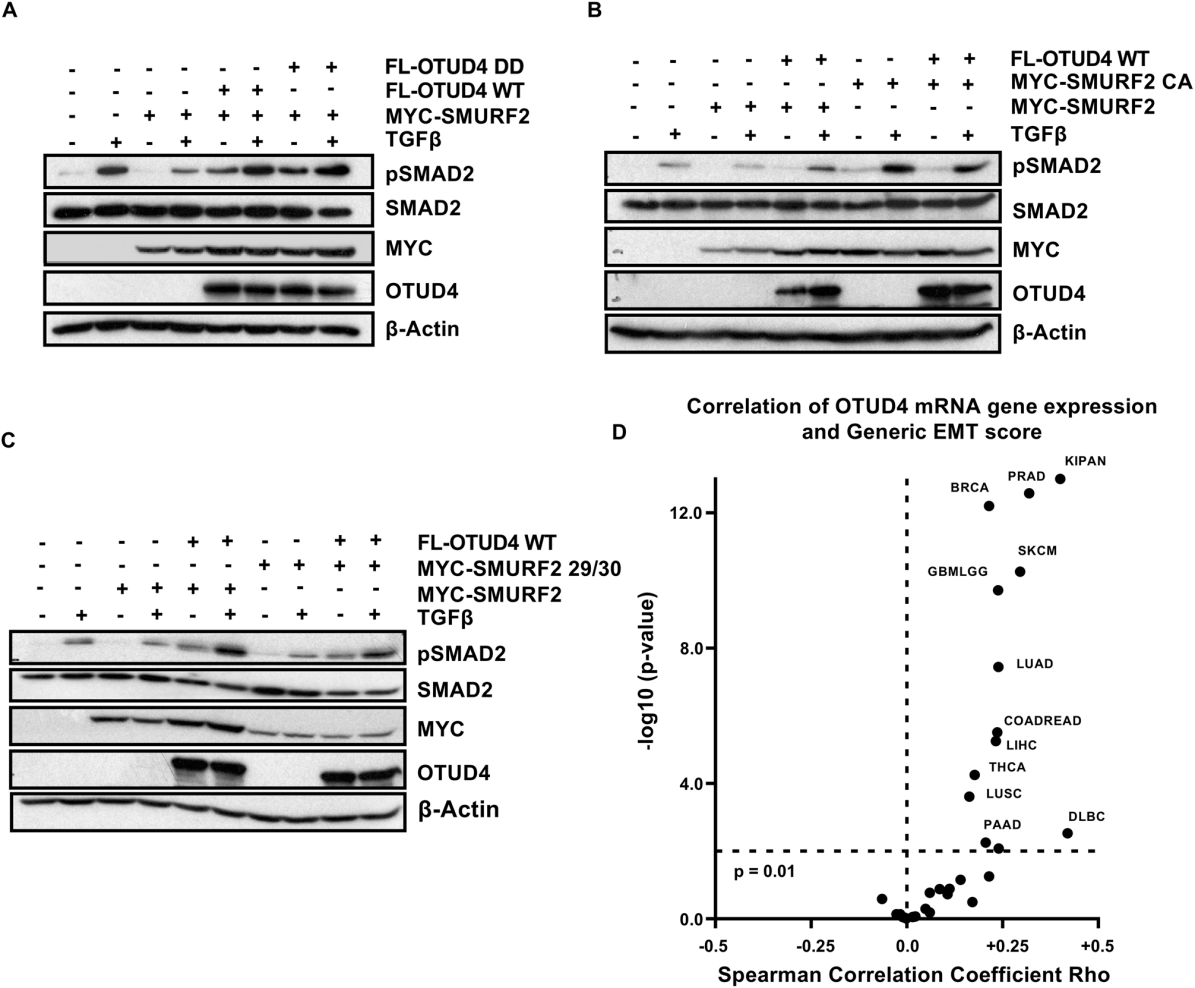
OTUD4 regulates SMURF2 auto-regulation. (**A**) HEK293T cells transfected with MYC-SMURF2 and either FLAG-OTUD4 or FLAG-OTUD4 DD. Cells were stimulated where indicated with TGFβ (100 pM) overnight before lysis. After 48 h cells were lysed and whole cell extracts were probed with indicated antibodies. β-Actin is used as the loading control. (**B**) HEK293T cells transfected with either MYC-SMURF2 or MYC-SMURF2 CA and FLAG-OTUD4. Cells were stimulated where indicated with TGFβ (100 pM) overnight before lysis. After 48 h cells were lysed and whole cell extracts were probed with indicated antibodies. β-Actin is used as the loading control. (**C**) HEK293T cells transfected with either MYC-SMURF2 or MYC-SMURF2 29/30 and FLAG-OTUD4. Cells were stimulated with TGFβ (100 pM) where indicated overnight before lysis. After 48 h cells were lysed and blotted precipitates were probed with indicated antibodies. β-Actin is used as the loading control. (**D**) Spearman’s analysis between OTUD4 mRNA expression and Generic EMT score across a TCGA pan-cancer dataset (n = 12,290). Full-length blots for (**A**,**B**,**C**) are shown in Supplementary Information.

Considering SMURF2’s role in modulating the TGFβ pathway, we investigated whether or not OTUD4 affects the ability of SMURF2 to downregulate TGFβ signalling as determined by phosphorylated SMAD2 levels. Figure 5A demonstrates that overexpression of SMURF2 mitigates the ability of TGFβ ligand to increase pSMAD2 levels, an effect annulled upon the co-expression of OTUD4. Interestingly, OTUD4 DD was also functional in restoring pSMAD2 levels suggesting that this phenomenon is independent of OTUD4’s deubiquitinase activity in the same manner as OTUD4-induced TβRI deubiquitination (Figs. 3C, 5A). Importantly, both OTUD4 and the catalytically inactive mutant OTUD4 DD significantly increased the expression levels of SMURF2 (Fig. 5A). This strongly supports the postulation that OTUD4 may negatively regulate the autocatalytic activity of SMURF2 resulting in SMURF2 stabilization.

In order to determine whether OTUD4 perturbs SMURF2’s catalytic activity rather than the inhibition of an external ligase, we employed a SMURF2 catalytically inactive mutant, SMURF2 C716A (SMURF2 CA). Unlike the results with wild type SMURF2, ectopic expression of OTUD4 was unable to induce an increase in SMURF2 CA levels highlighting the potential that the paradoxical stabilization of SMURF2 results from inhibition of SMURF2 autoubiquitination (Fig. 5B, Sup. Fig. 3A). To further confirm this, we co-transfected OTUD4 with the SMURF2 mutant FF29/30AA (SMURF2 29/30). These mutations preclude SMURF2’s ability to adopt a closed conformation, rendering it constitutively active, but highly unstable^11^. In line with our previous observations, co-expression of SMURF2 29/30 completely abolished the ability of OTUD4 to stabilize SMURF2 (Fig. 5C, Sup. Fig. 3B). Collectively, these results strongly suggest that OTUD4 may function to maintain SMURF2 in a closed, inactive and more stable conformation. It is the downregulation of SMURF2 activity by OTUD4 which may partially explain the decrease in TβRI ubiquitination levels.

### OTUD4 regulates epithelial mesenchymal transition

As TGFβ is a major regulator of epithelial-mesenchymal transition (EMT) we sought to determine if OTUD4 regulates EMT in cancer. To this end we probed the TCGA pan-cancer dataset (n = 12,290) and correlated OTUD4 expression with a generic EMT signature described in Tan et al.^32^. We found that OTUD4 expression significantly correlated (Spearman correlation, p < 0.01) with the generic EMT score in 12 of the 37 tumour types tested (Fig. 5D, Sup. Table 2). Within all 12 of these tumours the correlation was positive. Among the most significant tumours were renal cancer, prostate adenocarcinoma and breast cancer. Interestingly, when the TCGA pan-cancer dataset was analyzed as a whole, OTUD4 expression negatively correlated with the generic EMT signature. This suggests that while OTUD4 alters TGFβ signalling in the majority of cancers it only positively correlates with EMT in a relatively minority cancers highlighting the tissue specificity of OTUD4 and EMT in cancer plasticity.

## Discussion

In this report we demonstrate that OTUD4 is a potent deubiquitinating enzyme required for the augmentation of TGFβ signalling via TβRI stabilization at the plasma membrane. We can therefore add OTUD4 to the growing list of DUBs that have already been demonstrated to specifically regulate the TβR complex. DUBs that have already been reported to affect the TβR complex include: USP4, USP11, USP15, USP26, UCH37, and UCHL1^7,19,24,26,27,33^. The identification of a large number of DUBs regulating TβR levels is not altogether surprising as recent evidence has indicated that 18 DUBs have been demonstrated to effect EGFR kinetics and 12 DUBs have been demonstrated to effect c-MET levels^34,35^. The large number of deubiquitinating enzymes demonstrated to affect receptor kinetics highlights the importance of ubiquitin as a fundamental regulator of receptor processing and downstream signalling.

In terms of the TβR complex, the precise mechanisms by which ubiquitin regulates signalling and kinetics remains poorly understood. Currently there is a paucity of reports describing in detail ubiquitin’s role in TβR endocytosis and trafficking. In fact, only very recently has it been confirmed that TβR is trafficked by the ESCRT machinery to the lysosome for destruction^36^. This implies that the ubiquitin-mediated regulation of this trafficking machinery has implications with respect to TβR signalling and kinetics^28^.

Different ubiquitin chain topologies act as signals to regulate the outcome of various substrates. One of the most functionally well-characterized chain topologies, K48-linked, serves as the prototypical degradation signal, shunting ubiquitinated proteins to the proteasome for degradation^15^. K63-linked chains, on the other hand, perform a number of non-proteolytic functions including cellular signalling, endocytosis and intracellular trafficking^15,29^. A number of the DUBs mentioned above are able to remove ubiquitin chains, likely the K48-linked type, from TβR resulting in its stabilization^19,24,26^. Given that proteasome inhibitor MG132 did not rescue TβRI upon OTUD4 knockdown suggests that in the absence of ligand, the ubiquitin chain regulated by OTUD4 is unlikely to be K48-linked. In line with this notion, ectopic expression of OTUD4 did not enhance the stabilization of TβRI even though OTUD4 decreased the overall levels of incorporated ubiquitin. Interestingly, treatment with the lysosomal inhibitor chloroquine rescued TβRI levels in the presence of OTUD4 knockdown, suggesting that OTUD4 may function to regulate TβR endocytosis and/or trafficking rather than directly influencing its proteasome-mediated degradation. OTUD4 was initially described as a K48-specific DUB^21^ and indeed it has been reported to regulate the stability of anti-viral protein, MAVS, by removing K48-linked ubiquitin chains in a catalytically dependent manner^37^. Recent evidence however, has revealed that phosphorylation of OTUD4 can alter the enzymatic activity of the protein, permitting a K63-linkage specific deubiquitination of the Toll like receptor/ Interleukin-1 receptor (TLR/IL-1R) associated factor, MyD88^38^. OTUD4 has also been found to act downstream of MyD88 by negatively regulating TRAF6-mediated K63 autoubiquitinaiton^39^. Ultimately, it appears that OTUD4’s ability to regulate cellular function by removing either K29, K48 or K63 polyubiquitin chains is context dependent.

Ubiquitin has long been implicated in regulating the endocytosis of plasma membrane receptors and though this is a disputed issue, K63-linked ubiquitin chains are believed to play a role in this process^29–31,40,41^. The fact that ectopic expression of OTUD4 leads to an increase in plasma membrane levels of this receptor subunit lends support to the notion that OTUD4-regulated ubiquitin chains affects endocytosis. Figure 4E suggests that OTUD4 regulates K63-linked chains of TβRI, though we have only investigated this in the context of activated TβRI.

It is evident that the catalytic activity of OTUD4 also plays a fundamental role in regulating this signalling pathway. Importantly, clues alluding to the purpose of OTUD4 catalytic activity are only revealed upon the addition of TGFβ ligand or in the context of constitutively active TβRI. This is clearly observed in Fig. 1F where both wild type OTUD4 and OTUD4 catalytically inactive mutants are able to increase baseline levels of TGFβ activity but, upon the addition of TGFβ ligand, TGFβ-mediated transcription is severely impaired in cells expressing the OTUD4 catalytically inactive mutants. In the presence of the ligand, the catalytic activity of OTUD4 is important for maintaining TβRI at the plasma membrane (Fig. 4A). This may be due to the inability of OTUD4 DD to remove ubiquitin from activated TβRI as demonstrated in Fig. 4B. The fact that OTUD4’s deubiquitinase activity is only important for removing ubiquitinated chains in the context of receptor activation suggests that either activation of the receptor induces the attachment of different chain topologies from those attached in the non-activated context or the activated receptor is localized to a cellular compartment that is conducive for OTUD4’s direct deubiquitinase activity. It is possible that while removal of ubiquitin in the non-activated context may impede endocytosis, perhaps the removal of ubiquitin from an activated receptor by OTUD4 affects the process of recycling post-endocytosis, resulting in less TβRI being trafficked to the lysosome and more being recycled to the plasma membrane. Similar to this, USP8 has been shown to prevent the ubiquitination of Wnt signalling component, Smo, preventing its localization into early endosomes and increasing its presence at the plasma membrane^42^.

Whether or not OTUD4 is also phosphorylated when acting in these different compartments requires further investigation. Though OTUD4 complexes with TβRI, it is unlikely to be phosphorylated directly by this receptor subunit as the motif recognised by TβRI is typically SXS^5^. In contrast, the phosphorylation of OTUD4 occurs at a SXXE/D motif^38^ which is recognised by the kinase casein kinase 2 (CK2)^38,43^. CK2 has a large number of substrates from various cellular compartments^43^ suggesting that this kinase could feasibly influence OTUD4′s chain specificity both at the plasma membrane^44^ or post-endocytosis.

Our data points also to the possibility that OTUD4 might indirectly regulate the ubiquitination of TβRI by preventing the ligase SMURF2 from ubiquitinating the receptor. Though the action of SMURF2 has traditionally been believed to result in proteasomal degradation of its substrates, it has been reported that this ligase can conjugate K63 type chains, and thus potentially regulate endocytosis^6,29,45^. Interestingly, the ability of OTUD4 to negatively regulate SMURF2 appears to be independent of its catalytic activity (Fig. 5A). How OTUD4 exerts this effect on SMURF2 remains unclear. One possible theory is that OTUD4 impedes the ability of SMAD7 to bind to SMURF2 and thereby limiting access of SMURF2 to the TβR complex. The observation that OTUD4 can still induce SMAD2 phosphorylation in the presence of SMURF2 29/30, despite not being able induce the inactive state of the ligase, indicates that this may be a potential mechanism of action.

It has recently been reported that OTUD4 can also act as a scaffold to bring DUBs USP7 and USP9X to remove ubiquitin from the oxidative demethylase ALKBH3 resulting in its stabilization^46^. Likewise, USP9X, but not USP7, bound to the TβR complex resulting in TβRII stabilization but we were unable to determine if these effects were dependent upon OTUD4 (data not shown). Furthermore, we were unable to verify if the effects of USP9X on the TβR complex was mitigated by SMURF2 inhibition. If indeed OTUD4-bound USP9X can affect TβR deubiquitination, it may explain both the catalytic and non-catalytic functions of OTUD4 in TGFβ regulation.

Given the fundamental cellular processes regulated by TGFβ, a critical understanding of the receptor activation, internalization and degradation is required to potentially target the receptor complex in cancer. Targeting the TGFβ pathway has become a promising therapeutic strategy in certain cancers^47^. An understanding, therefore, of the components of the TGFβ pathway operating in cancer is integral in defining novel predictive biomarkers to direct the use of therapeutic compounds. We have provided evidence of OTUD4′s ability to regulate the TGFβ pathway in cancer and in certain contexts influences EMT. The current literature alludes to the possibility of OTUD4 being a tumour suppressor in lung cancer, liver cancer and breast cancer^48–50^. Nevertheless, in situations where TGFβ drives tumor progression, such as glioblastoma^19^, OTUD4 is likely to promote oncogenesis. Ultimately, the identification of OTUD4 adds greater resolution to our knowledge surrounding the cell’s ability to regulate the TGFβ pathway and defines OTUD4 as a biomarker for TGFβ activity, with its expression acting in proxy to reveal the intrinsic activity of this cancer-relevant pathway.

## Methods

### Western blotting and quantification

Cells were lysed in solubilizing buffer (50 mM Tris pH 8.0, 150 mM NaCl, 1% NP-40, 0.5% deoxycholic acid, 0.1% SDS, 200 µM Sodium Vanadate, 1 µM magnesium chloride, 50 mM sodium fluoride, 25 mM β-glycerol phosphate), supplemented with protease inhibitors (Complete; Roche). Whole cell extracts were then separated on 7–12% SDS-Page gels and transferred to polyvinylidene difluoride membranes (Millipore). Before antibody probing, membranes were blocked with bovine serum albumin except when antibody probing was for phospho-SMAD2, in which case the membrane was blocked in milk. Blots were then incubated with an HRP-linked second antibody and signal was detected with chemiluminescence (Pierce) using film and developed using film developer (Konica Minolta). Image-J software (https://imagej.nih.gov/ij/) was used to quantify resultant Western blots.

### Plasmids and antibodies

The DUB knockdown library vectors were generated by annealing the individual oligonucleotide primer pairs and cloning them into pRETROSUPER (pRS) as described in Brummelkamp et al.^20^. The bacterial colonies of each DUB hairpin were then pooled and used for plasmid preparation. For OTUD4 knockdown sequences are as follows: (A) 5′ CAGAGAGAAATTTGAAGCGT 3′; (B) AGTATAAAGAAAGCTCTGCT; (C) 5′ AAGTGCCCTTTCTCTTATGT 3′; (D) 5′ AAGAAAGCTCTGCTATGTGT 3′. OTUD4 knockdown sequences utilized for stable expression with lentivirus infection are as follows: KD1 5′ GCGTTTATAGAAGGATCATTT 3′; KD2 5′ GAGATTGGACCGCCGACATTT 3′; KD6 5′ CACTATAGATTCCAAACATAA 3′. Human FLAG-OTUD4 was purchased from MRC Protein phosphorylation and Ubiquitylation unit (#DU22035). Generation of catalytically inactive OTUD4 mutants were generated by site directed mutagenesis as described in Papa et al.^51^. The following plasmids were purchased form addgene: MYC-SMURF2 (#13678), MYC-SMURF2 C716A (#13678), MYC-SMURF2 FF29/30AA (#24604), HA-Ubiquitin (#17608), HA-Ubiquitin K11 (#22901), HA-Ubiquitin K27 (#22902), HA-Ubiquitin K29 (#22903), HA-Ubiquitin K48 (#17605), HA-Ubiquitin K63 (#17606). TGFβRI, FLAG-TβRI C.A. and FLAG-SMAD7 were kind gifts from Joan Seoane. CAGA luciferase and SV40-Renilla were kind gifts from Rene Bernards. Additional cloning information will be given upon request. The following antibodies were purchased from Cell Signaling Technologies: anti-p-SMAD2 (#3101), anti-SMAD2 (#3103), anti-β-TUBULIN (#2128). The following antibodies were purchased form Santa Cruz Biotechnology: anti-HA (#sc805 or #sc57592), anti-MYC (#sc40 or #sc78), anti-TβRI (#sc398 or #sc399). The following antibodies were purchased from Sigma-Aldrich: anti-FLAG (#F7425) and anti-β-ACTIN (#A1978). Anti-OTUD4 (#ab106971) was purchased from Abcam, Anti-SMAD7 (#MAB2029) was purchased from R and D Systems. Anti-TβRI (#AHO1552) (for confocal immunofluorescence microscopy) was purchased from Thermo Fisher Scientific. The following conjugated secondary antibodies used for confocal immunofluorescence microscopy were purchased from Thermo Fisher Scientific: Alexa Fluor 647 (#A-21244) and Alexa Fluor 555 (#A-31570). Phalloidin CruzFluor 488 (#sc363791) was purchased from Santa Cruz Biotechnology.

### Cell culture and transient transfections

HEK293T, MCF7 and MDA-MB-231 cells were cultured in Dulbecco's modified Eagle medium (DMEM- High glucose with L-glutamine (Hyclone)) supplemented with 10% fetal bovine serum (Hyclone), 1% sodium pyruvate (Hyclone) and 1% Penicillin/Streptomycin (Gibco). HEK293T cells were divided in 10-cm dishes 1 day prior to transfection. Sub-confluent cells were transfected using the calcium phosphate transfection method^52^. Cells were incubated overnight and washed twice in PBS. Lysates were collected 48–72 h post transfection. When appropriate, TGFβ (100 pm; R&D), SB431542 (1 µM: Tocris), MG132 (5μM or 10 µM; Calbiochem) or Chloroquine (400 µM; Sigma-Aldrich) were added. Lipofectamine 2000 (Thermo Fisher Scientific) was used to transfect cells for confocal immunofluorescent microscopy. Briefly, the appropriate DNA was mixed together with 15 µl of lipofectamine 2000 in supplement-free DMEM for 30 min. After incubation period, the mixture was added to HEK293T cells.

### Lentivirus transduction

A 10 cm dish of HEK293T was transfected with lentivirus packaging vector DNA constructs (pMDLg/pRSV-REV/pMD2.G) as well either one of three shRNA hairpins specific to OTUD4 cloned within the vector pLKO.1. An shRNA targeting GFP within the pLKO.1 vector (lenti GFP) was transfected along with the lentivirus packaging vector DNA constructs to act as the negative control. The day after transfection, each plate was washed twice with PBS and 6mls of fresh DMEM media was added. The following day the supernatant from each 10 cm dish (which now contained virus) was collected and placed within a 10 ml syringe and then filtered through a 0.45 µm filter. Either HEK293T MCF7s, or MDA-MD-231s were incubated overnight with virus (between 400 µl and 12.5 µl of virus to a well of a 6-well plate). Polybrene (Sigma-Aldrich) was added to the media of recipient cells just before incubation with virus.

### Luciferase assays

Luciferase assays were performed in a 12-well plate using the Dual luciferase system (Promega). CAGA-luciferase vector (200 ng per well) and SV40-Renilla (40 ng per well) was transfected in the presence of FLAG-OTUD4 (400 ng per well), or either FLAG OTUD4 mutants (400 ng per well), or a control vector. For loss-of-function experiments, CAGA-luciferase vector (200 ng per well) and SV40-Renilla (40 ng per well) was co-transfected with 1.5 μg per well of relevant pRS control vector or pRS- OTUD4 knockdown vectors. After 72 h 100 pM TGFβ was added in the presence of DMEM (0% FCS) and luciferase counts were measured approximately 16 h later using a Sirius Luminometer (Berthold).

### Immunoprecipitation and in vivo deubiquitination assay

For coimmunoprecipitation experiments cells were lysed in ELB (0.25 M NaCL, 0.5% NP-40, 50 mM HEPES [pH 7.3]) supplemented with proteasome inhibitors (Complete; Roche). Cell lystates (500 μg to 1 mg) were incubated overnight with 1 μg of the indicated antibodies conjugated. Subsequently the lysates were then incubated for up to 6 h with protein A or protein G sepharose beads (GE Healthcare), washed three times in ELB buffer and separated out on SDS-PAGE gels. For in vivo ubiquitination experiments TβRI (5 μg) or TβRII (5 μg) was co-transfected with HA-Ubiquitin (5 μg) and FLAG-OTUD4 (5 μg), FLAG-OTUD4 DD (5 μg), or a control vector. For loss-of-function experiments FLAG-TβRI C.A. (2 μg) were co-transfected with HA-Ubiquitin (5 μg) and pRS OTUD4 B, pRS OTUD4 C (10 μg) or control vector. For the FLAG-TβRI C.A. experiment, after 72 h MG132 (5 μM) was added, incubated overnight, and cells were lysed in ELB buffer.

### Quantitative real time PCR

Cells were collected, washed twice in PBS and RNA was isolated using GeneJet RNA extraction kit (Thermo-Scientific) qRT was performed using specific mRNA primers and SYBR green chemistry (Applied Biosystems). Reactions were carried out on a ABI 7900 or 7500 FAST sequence detector (Applied Biosystems). Relative mRNA values are calculated by the ∆∆Ct method. GAPDH was used as internal normalization controls where specified. The following qPCR primers were used SMAD7: 5′‐AAA CAG GGG GAA CGA ATT ATC‐3′, 5′‐ACC ACG CAC CAG TGT GAC‐3′; PAI-1; 5′-GTGTTTCAGCAGGTGGCGC-3′, 5′-CCGGAACAGCCTGAAGAAGTG-3′ ; CTGF: 5′-TAGGCTTGGAGATTTTGGGA-3′, 5′-GGTTACCAATGACAACGCCT-3′; GAPDH: 5′‐AAC AGC GAC ACC CAC TCC TC‐3′, 5′‐CAT ACC AGG AAA TGA GCT TGA C‐3′.

### Biotinylation

Biotin (Thermo Scientific) was resuspended in PBS at a concentration of 0.25 mg/ml. 5 mls was added to cells in a 10 cm dish and incubated at 4 °C on a shaking incubator for 40 min. For each sample, after 40 min, 500 µl of quenching solution was added to the biotin supernatant and the entire supernatant was then removed from the cells and put into a 50 ml centrifuge tube. Cells were incubated in 6 mls of quenching solution on ice for 5 min before being scraped from the plate and collected in the same 50 ml centrifuge tube along with the biotin solution. Cells were pelleted and then washed with TBS. Afterward the cells were resuspended in 500 μl of ELB. Lysate was then sonicated using the Diagenode Biorupter Plus. Settings of the Biorupter Plus were as follows: low power, 3 × 5 s bursts with a 5 s delay in-between. After sonication, lysates were incubated on ice for 30 min and vortexed every 5 min for 5 s. Afterward, lysates were centrifuged at 10,000*g* for 2 min at 4 °C (Tomy MX-305 high speed centrifuge). The lysates (500 µg of protein) were incubated with NeutrAvidin agarose resin (Thermo Fisher Scientific) and rotated at 4 °C overnight. The NeutrAvidin agarose resin was then washed three times in ELB buffer and separated out on SDS-PAGE gels. Immunoblotting for proteins of interest was then performed.

### Confocal immunofluorescence microscopy

HEK293T cells were transfected with cDNA of interest using lipofectamine 2000 (Thermo Fisher Scientific). Cells were seeded onto glass coverslips (12 mm) that had been coated for 30 min with Poly-d-Lysine (0.05 mg/ml). After approximately 48 h from the point of transfection cells were stimulated with TGFβ (100 pM) for 1 h. Cells were then fixed in 4% formaldehyde for 15 min. Cells were then permeabilized in 0.2% Triton X-100 (in PBS) for 10 min. Cells were blocked in 5% BSA( in PBS) for a minimum of 45 min. Cells were incubated in primary antibodies (dilution factor 1:100 in 5% BSA (in PBS)) for 1 h at room temperature. Cells were then incubated with fluorescently conjugated secondary antibodies and Phalloidin 488 (1:1000 dilution in 5% BSA (in PBS)) for approximately 1 h in the dark. Coverslips were then mounted on Polysine glass slides (Thermo Fisher Scientific) with mounting media infused with DAPI (Vectashield). Subsequently coverslips were subjected to confocal microscopy using Zeiss LSM 880 Airy Scan.

### Gene expression analysis

To study the correlation of OTUD4 mRNA gene expression and TGFβ signalling or epithelial-mesenchymal transition (EMT) in various cancers, FPKM-normalized gene expression data from TCGA cohorts were downloaded from Broad GDAC Firehose (https://gdac.broadinstitute.org/; last accessed Dec 2019; data version 2016_01_28)^53^. To estimate the TGFβ signalling activity in each sample, GSVA v1.28.0 was used to project TGFβ signature from Msigdb v6.1 hallmark collection on the TCGA dataset^54,55^. On the other hand, to estimate EMT phenotype, EMT scoring was performed on each sample using gene signature and method described in Tan et al.^32^.

### Statistical analysis

Correlation analyses were conducted using Matlab R2016b version 9.1.0.960167, statistics and machine learning toolbox version 11.0 (MathWorks; Natick, MA, USA). Volcano plots were made using GraphPad Prism version 5.04 (GraphPad Software, La Jolla, CA, USA).

Student’s T-test was performed in Microsoft Excel for luciferase and Western blot quantifications, p ≤ 0.05 was considered statistically significant.

## Supplementary information

Supplementary Information 1.

Supplementary Information 2.

Supplementary Information 3.
