# Supplementary material for: OTUD4 enhances TGFβ signalling through regulation of the TGFβ receptor complex
Source: Sci Rep. 2020 Sep 24;10:15725. doi: 10.1038/s41598-020-72791-0 (PMC7519109; doi:10.1038/s41598-020-72791-0)
Supplement: Supplementary file 1 — Supplementary Information 1. [file 41598_2020_72791_MOESM1_ESM.docx]

**Figure Supplementary 1**

(A) HEK293T cells stably expressing OTUD4 knockdown shRNA 1 (KD1), 2 (KD2), or relevant controls (shGFP). *OTUD4* mRNA levels relative to GAPDH are shown as evaluated by quantitative real-time PCR. Data are shown as the mean ± SD of triplicate samples from a representative experiment performed three times. (B) Schematic for OTUD4 showing the location of the catalytic triad. (C) HEK293T cells stably expressing OTUD4 knockdown shRNA 1 (KD1) and knockdown shRNA 2 (KD2). Cells were stimulated where indicated with TGFβ (100 pM) overnight before lysis. Cells were subsequently lysed and whole cell extracts were probed with indicated antibodies. β-Actin is used as the loading control. (D) MCF7 cells stably expressing OTUD4 knockdown shRNA 1 (KD1), knockdown shRNA 2 (KD2) and knockdown shRNA 6 (KD6). Cells were stimulated where indicated with TGFβ (100 pM) overnight before lysis. Cells were subsequently lysed and whole cell extracts were probed with indicated antibodies. β-Actin is used as the loading control (E) MDA-MB-231 cells stably expressing OTUD4 knockdown shRNA 1 (KD1) and knockdown shRNA 2 (KD2). Cells were stimulated where indicated with TGFβ (100 pM) overnight before lysis. Cells were subsequently lysed and whole cell extracts were probed with indicated antibodies. β-Actin is used as the loading control. Full-length blots for all relevant panels are shown in Supplementary Information.

**Figure Supplementary 2**

Confocal microscopy images of HEK293T cells transfected with TβRI, FLAG-OTUD4 and FLAG-OTUD4 DD as indicated. Cells were stimulated as indicated with TGFβ (100 pM) for 1 hour. Cells are stained with various antibodies as indicated. Phalloidin is a cytoplasmic marker that was included to denote the extent of each cell. White arrows indicate TβRI at the plasma membrane. Scale bar is 20 µm.

**Figure Supplementary 3**

A) Quantification of SMURF2 levels represented in 5B. Data are shown as the mean ± SD of 2 independent experiments. * P<0.05 as determined by Student’s T-Test. B) Quantification of SMURF2 levels represented in 5C. Data are shown as the mean ± SD of 3 independent experiments. * P<0.05 as determined by Student’s T-Test.

**Supplementary Table 1**

Spearman’s analysis between OTUD4 mRNA expression and TGFβ enrichment score (Hallmark geneset) score across a TCGA pan-cancer dataset (n= 12,290) and separated into individual tumour types.

**Supplementary Table 2**

Spearman’s analysis between OTUD4 mRNA expression and Generic EMT score across a TCGA pan-cancer dataset (n= 12,290) and separated into individual tumour types.
