# Supplementary material for: OTUD4 enhances TGFβ signalling through regulation of the TGFβ receptor complex
Source: Sci Rep. 2020 Sep 24;10:15725. doi: 10.1038/s41598-020-72791-0 (PMC7519109; doi:10.1038/s41598-020-72791-0)
Supplement: Supplementary file 2 — Supplementary Information 2. [file 41598_2020_72791_MOESM2_ESM.pptx]

## Slide 1
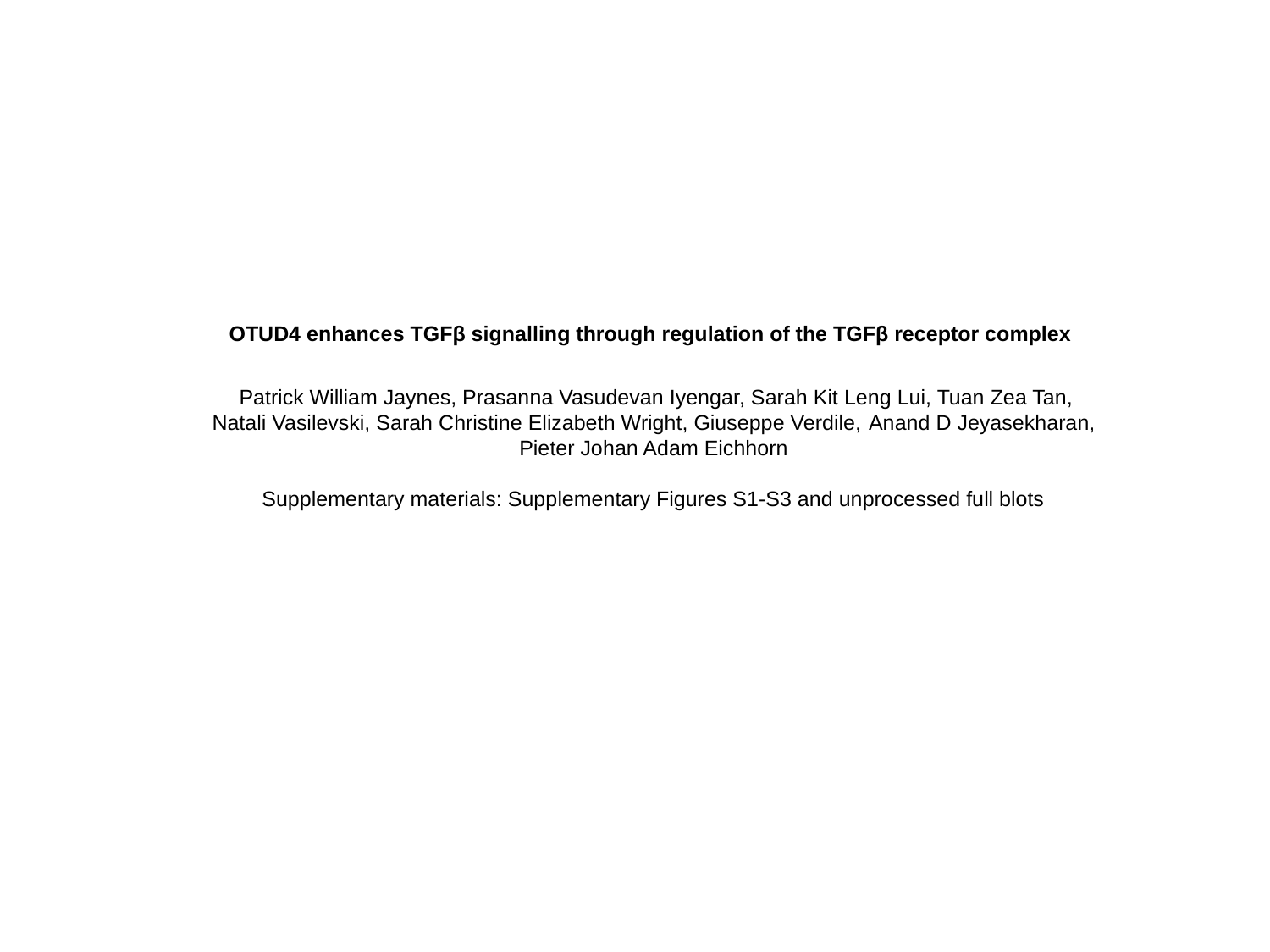

OTUD4 enhances TGFβ signalling through regulation of the TGFβ receptor complex
 Patrick William Jaynes, Prasanna Vasudevan Iyengar, Sarah Kit Leng Lui, Tuan Zea Tan, Natali Vasilevski, Sarah Christine Elizabeth Wright, Giuseppe Verdile, Anand D Jeyasekharan, Pieter Johan Adam Eichhorn
Supplementary materials: Supplementary Figures S1-S3 and unprocessed full blots

## Slide 2
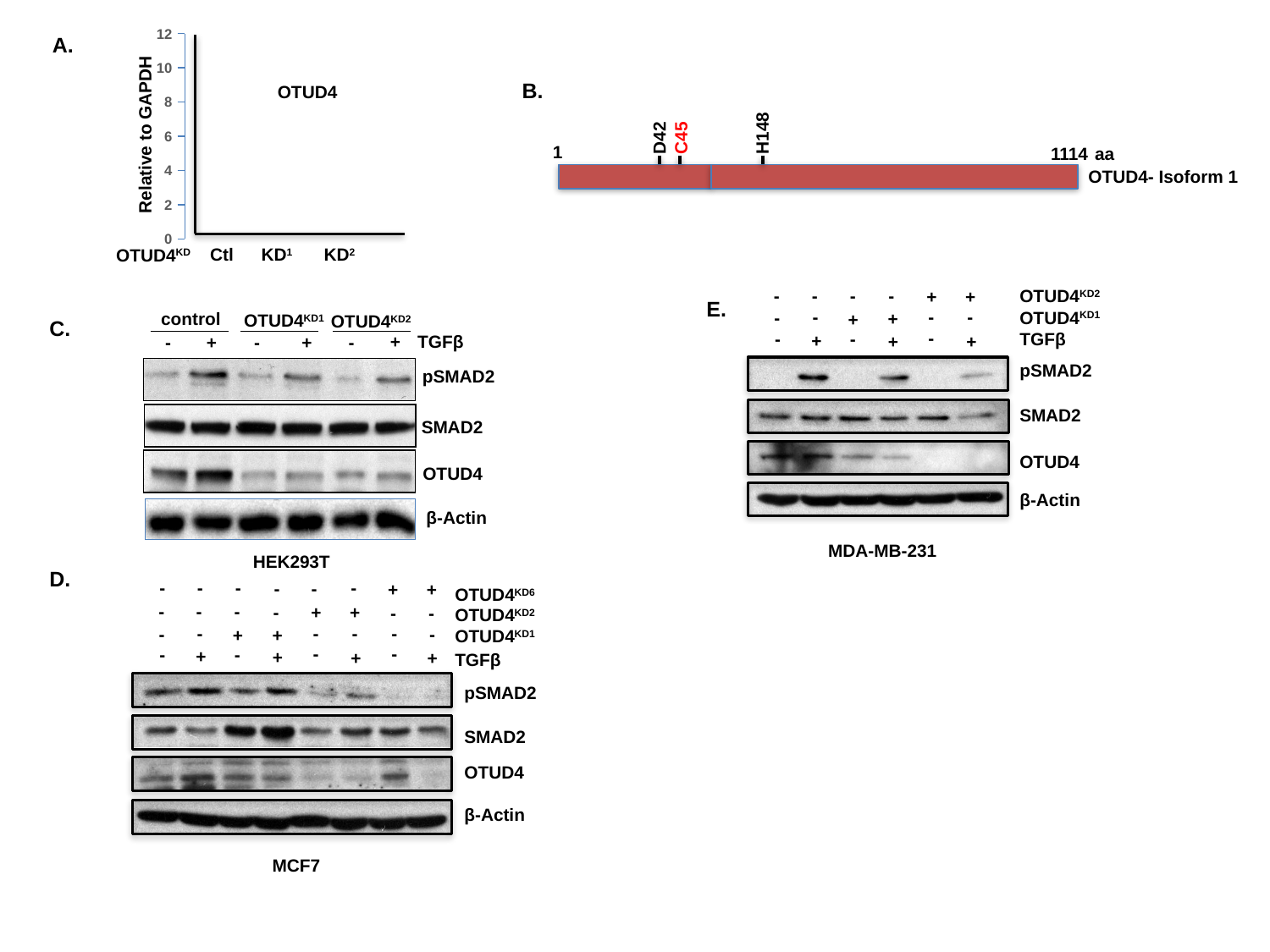

### Chart
| Category | |
|---|---|A.
B.
OTUD4
Relative to GAPDH
D42
C45
H148
 1
aa
 1114
OTUD4- Isoform 1
KD2
KD1
Ctl
OTUD4KD
-
-
-
-
OTUD4KD2
+
+
-
-
-
OTUD4KD1
-
+
+
-
-
TGFβ
-
+
+
+
pSMAD2
SMAD2
OTUD4
β-Actin
E.
control
OTUD4KD1
OTUD4KD2
TGFβ
+
-
-
+
+
-
pSMAD2
SMAD2
OTUD4
C.
β-Actin
HEK293T
MDA-MB-231
D.
-
-
-
-
-
-
+
+
-
-
-
-
+
+
-
-
-
-
-
-
-
-
+
+
-
-
-
-
+
+
+
+
β-Actin
OTUD4KD6
OTUD4KD2
OTUD4KD1
TGFβ
pSMAD2
SMAD2
OTUD4
MCF7

## Slide 3
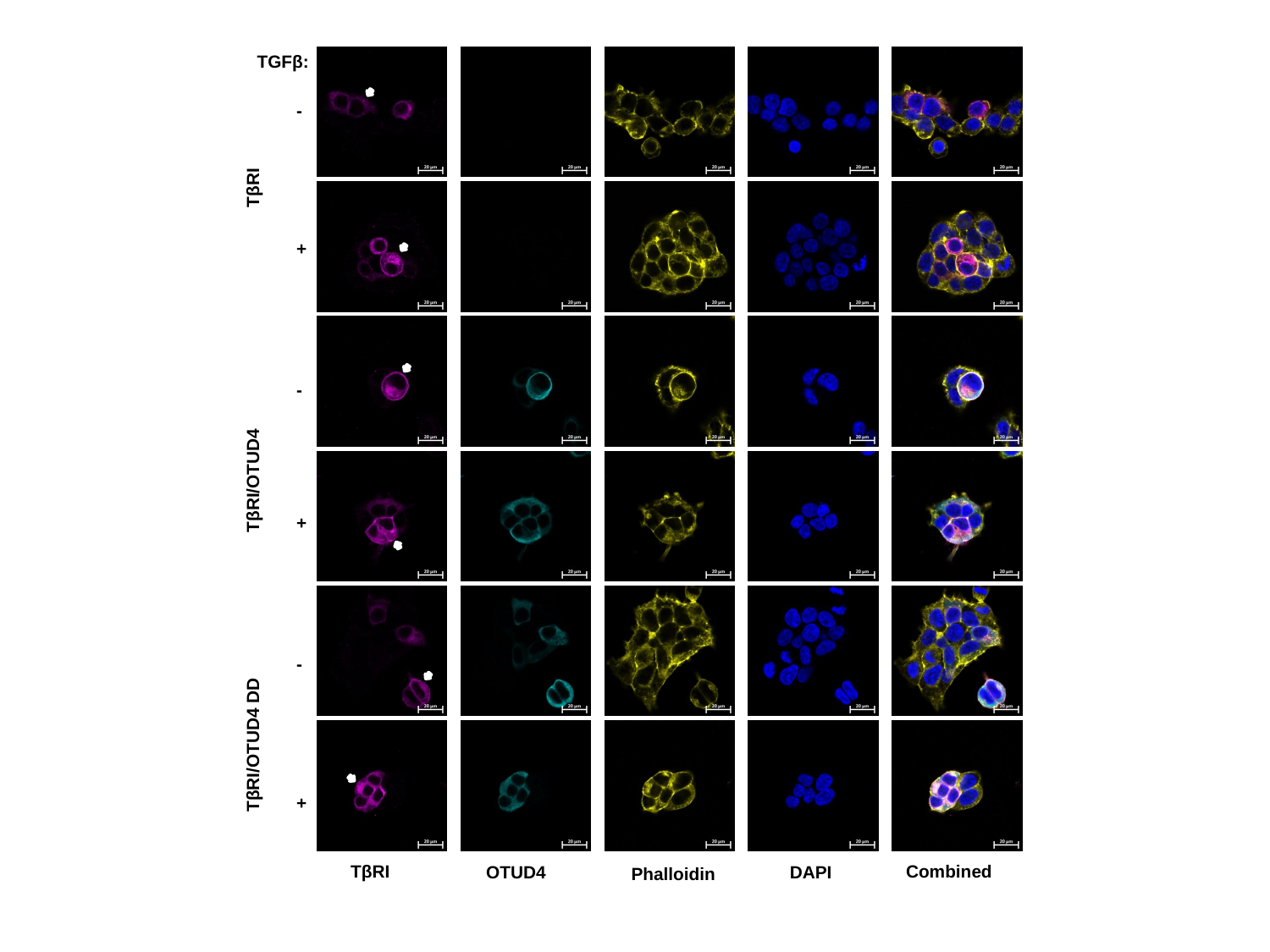

TGFβ:
-
TβRI
+
-
TβRI/OTUD4
+
-
TβRI/OTUD4 DD
+
Combined
TβRI
OTUD4
DAPI
Phalloidin

## Slide 4
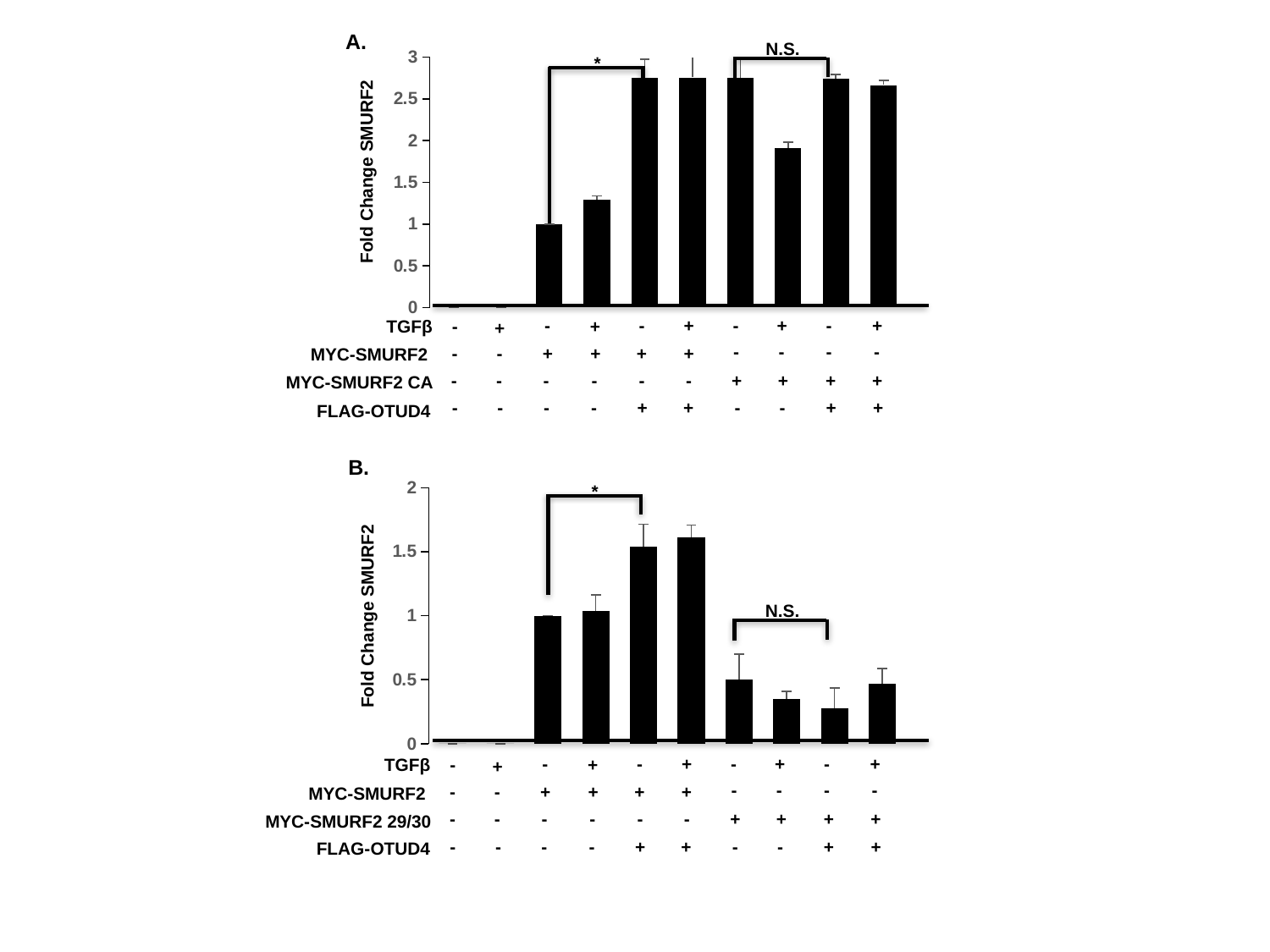

A.
N.S.
### Chart
| Category | |
|---|---|*
Fold Change SMURF2
-
-
+
-
+
-
+
+
-
TGFβ
+
-
-
-
-
-
-
+
+
+
+
MYC-SMURF2
-
-
-
-
-
-
+
+
+
+
MYC-SMURF2 CA
-
-
-
-
+
+
-
-
+
+
FLAG-OTUD4
B.
### Chart
| Category | |
|---|---|*
N.S.
Fold Change SMURF2
-
-
+
-
+
-
+
+
-
TGFβ
+
-
-
-
-
-
-
+
+
+
+
MYC-SMURF2
-
-
-
-
-
-
+
+
+
+
MYC-SMURF2 29/30
-
-
-
-
+
+
-
-
+
+
FLAG-OTUD4

## Slide 5
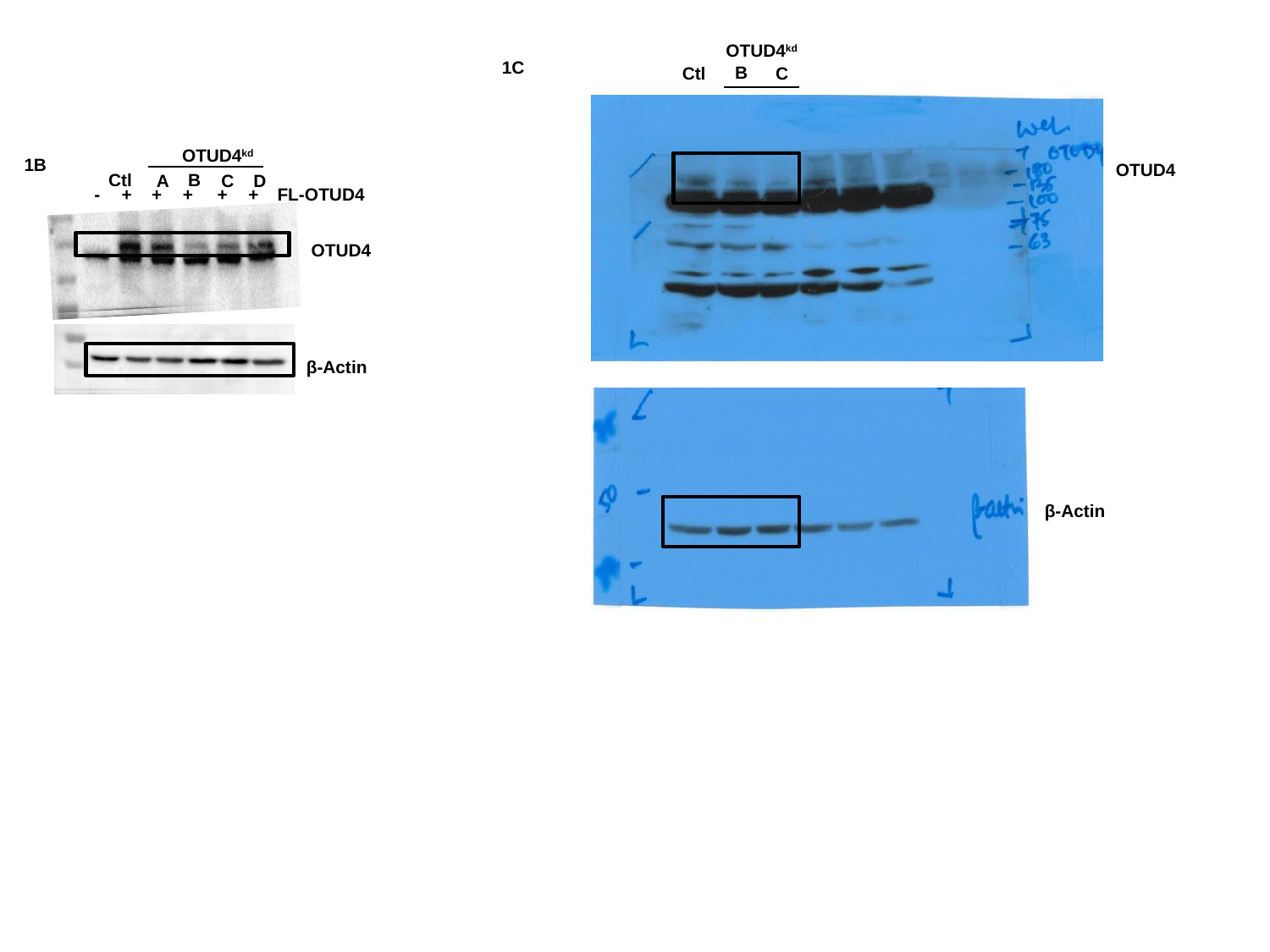

OTUD4kd
1C
B
Ctl
C
OTUD4kd
1B
OTUD4
Ctl
B
A
C
D
-
+
+
+
+
+
FL-OTUD4
OTUD4
β-Actin
β-Actin

## Slide 6
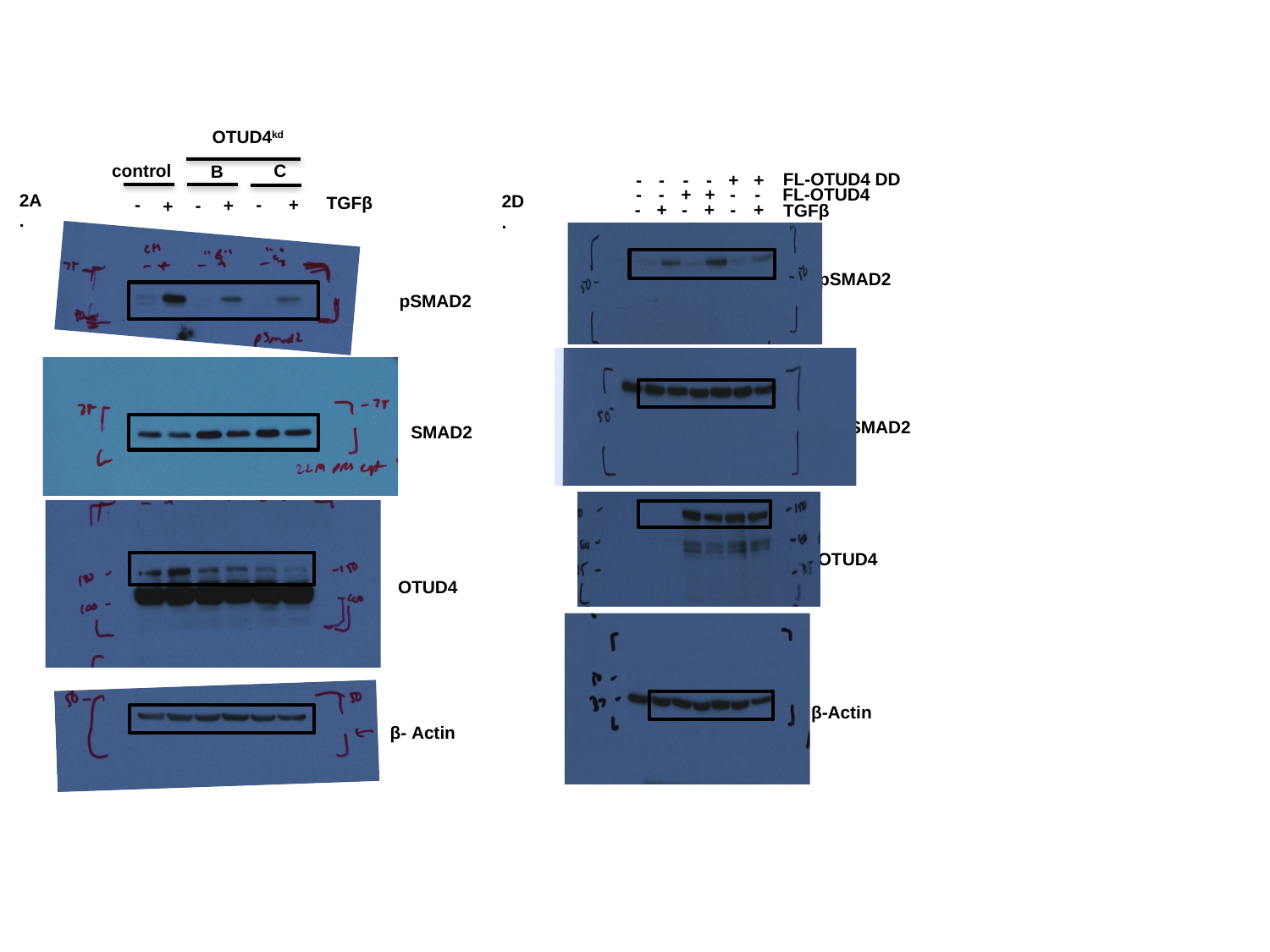

OTUD4kd
control
C
B
FL-OTUD4 DD
-
-
-
-
+
+
FL-OTUD4
-
-
+
+
-
-
2A.
2D.
TGFβ
+
-
-
+
-
+
-
+
-
+
-
+
TGFβ
pSMAD2
pSMAD2
SMAD2
SMAD2
OTUD4
OTUD4
β-Actin
β- Actin

## Slide 7
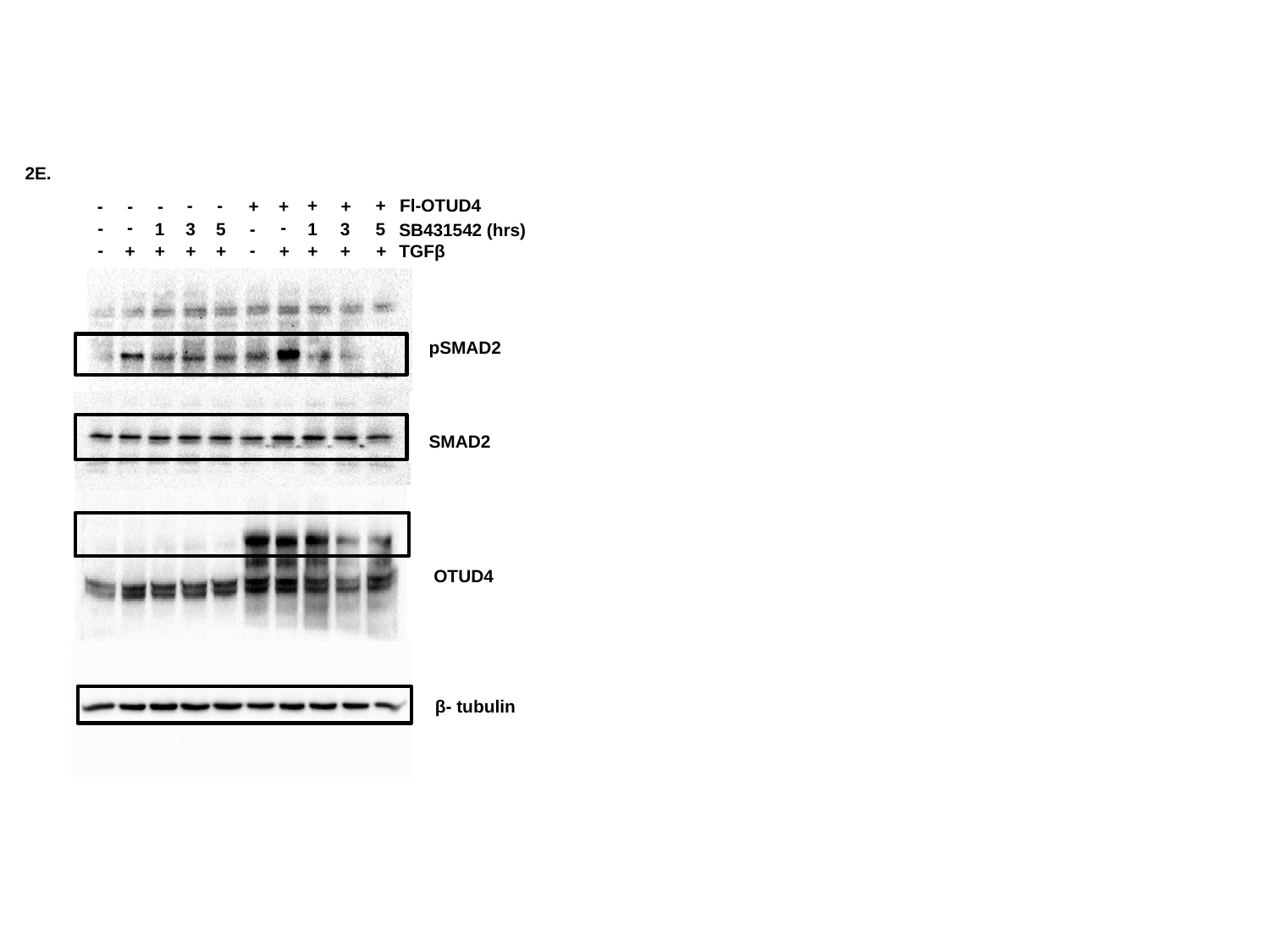

2E.
Fl-OTUD4
+
+
-
-
+
+
-
-
-
+
-
-
-
-
1
3
5
1
3
5
SB431542 (hrs)
-
-
+
+
+
+
+
+
+
+
TGFβ
pSMAD2
SMAD2
OTUD4
β- tubulin

## Slide 8
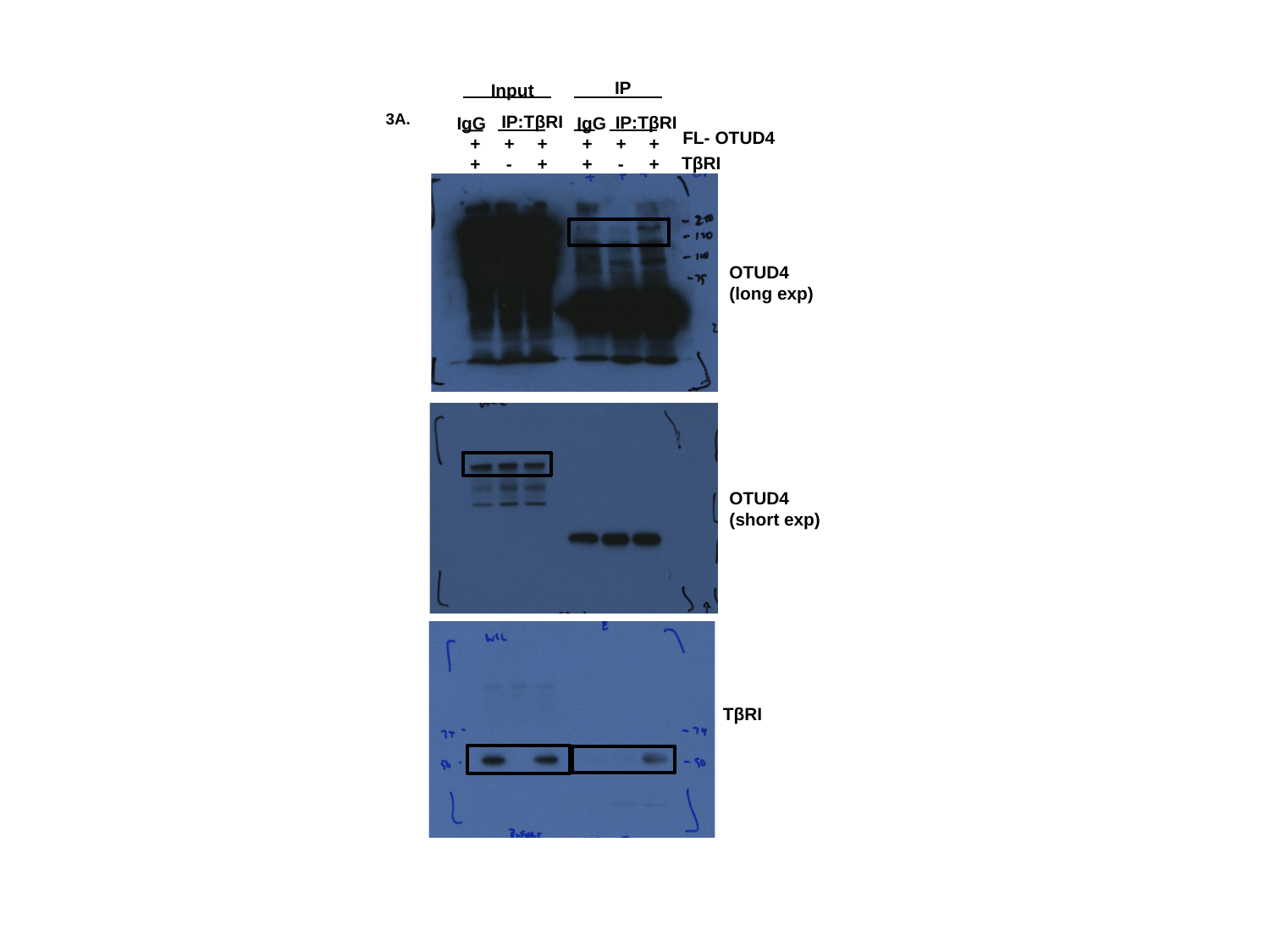

IP
Input
3A.
IP:TβRI
IP:TβRI
IgG
IgG
FL- OTUD4
+
+
+
+
+
+
TβRI
+
-
+
+
-
+
OTUD4 (long exp)
OTUD4
(short exp)
TβRI

## Slide 9
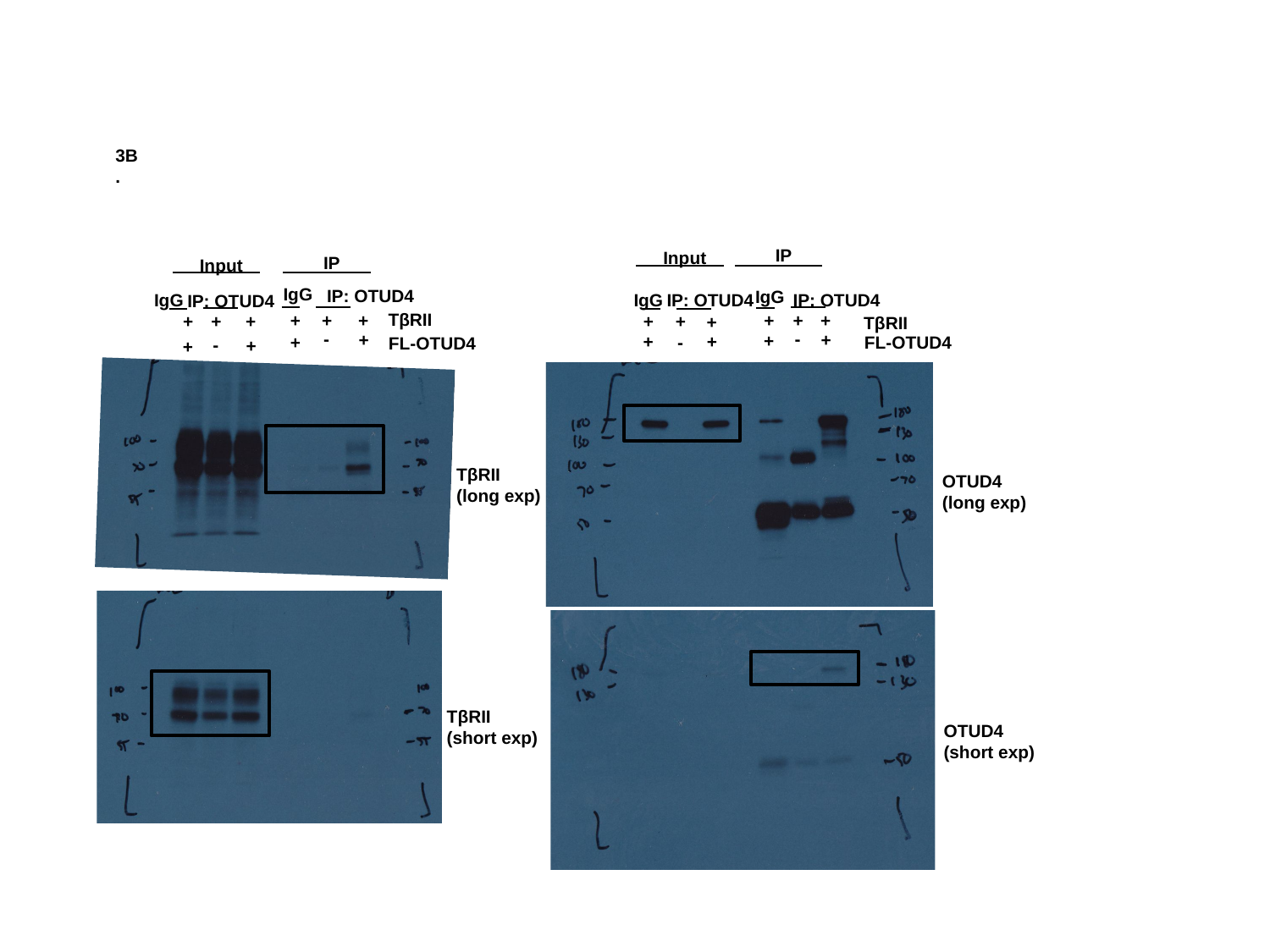

3B.
IP
Input
IP
Input
IgG
IP: OTUD4
IgG
IP: OTUD4
IgG
IP: OTUD4
IgG
IP: OTUD4
TβRII
+
+
+
+
+
+
+
+
+
+
+
+
TβRII
-
-
+
+
+
+
+
-
FL-OTUD4
+
FL-OTUD4
-
+
+
TβRII
(long exp)
OTUD4 (long exp)
TβRII
(short exp)
OTUD4 (short exp)

## Slide 10
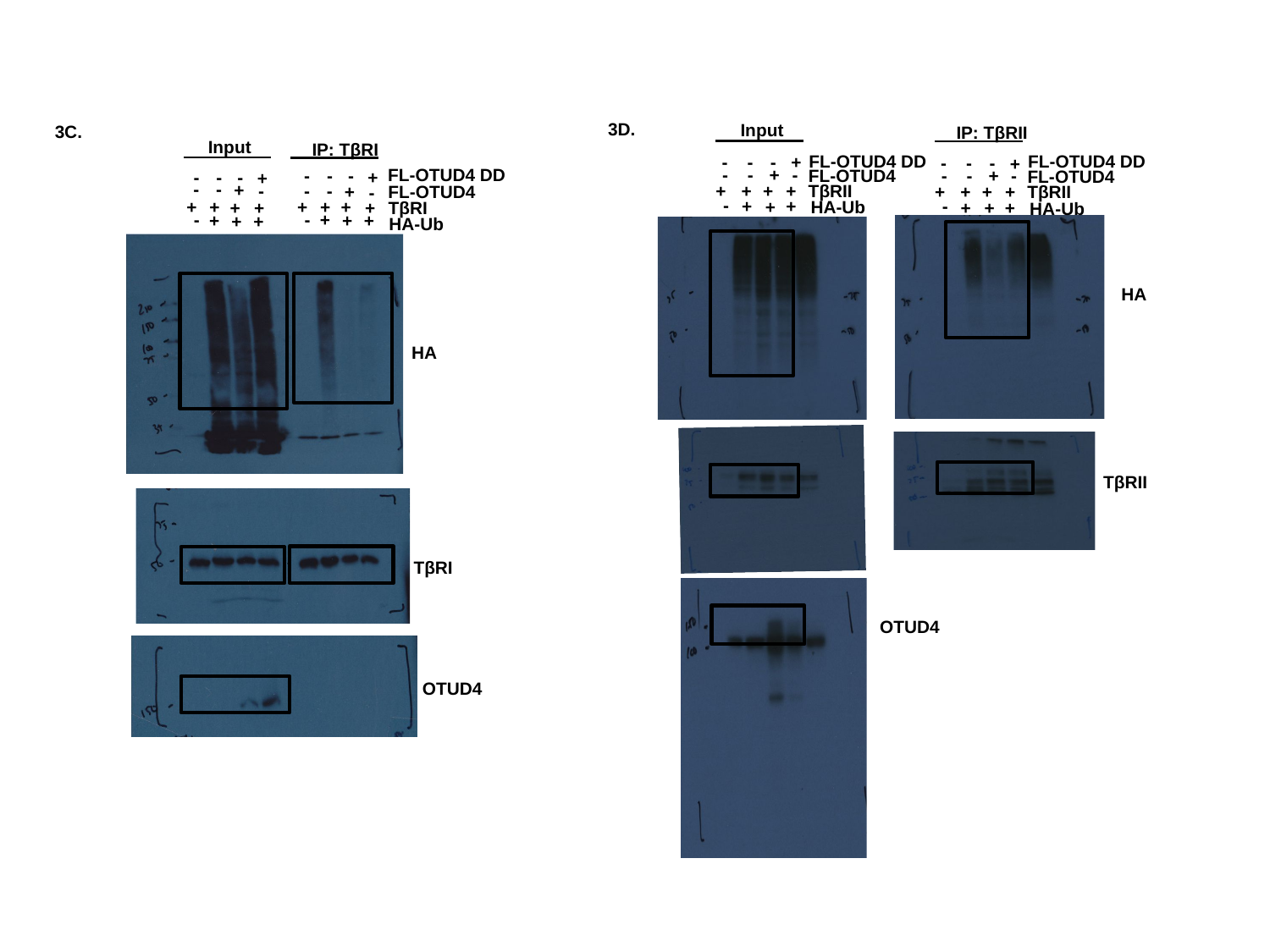

3D.
Input
3C.
IP: TβRII
Input
IP: TβRI
FL-OTUD4 DD
FL-OTUD4 DD
-
-
-
+
-
-
-
+
-
-
+
-
FL-OTUD4 DD
FL-OTUD4
-
-
-
-
-
+
-
FL-OTUD4
+
-
-
-
+
-
-
+
+
+
+
+
-
-
TβRII
-
+
+
+
+
+
FL-OTUD4
TβRII
-
-
-
+
+
+
+
+
+
+
HA-Ub
+
+
+
+
+
+
TβRI
+
HA-Ub
-
-
+
+
+
+
+
+
HA-Ub
HA
HA
TβRII
TβRI
OTUD4
OTUD4

## Slide 11
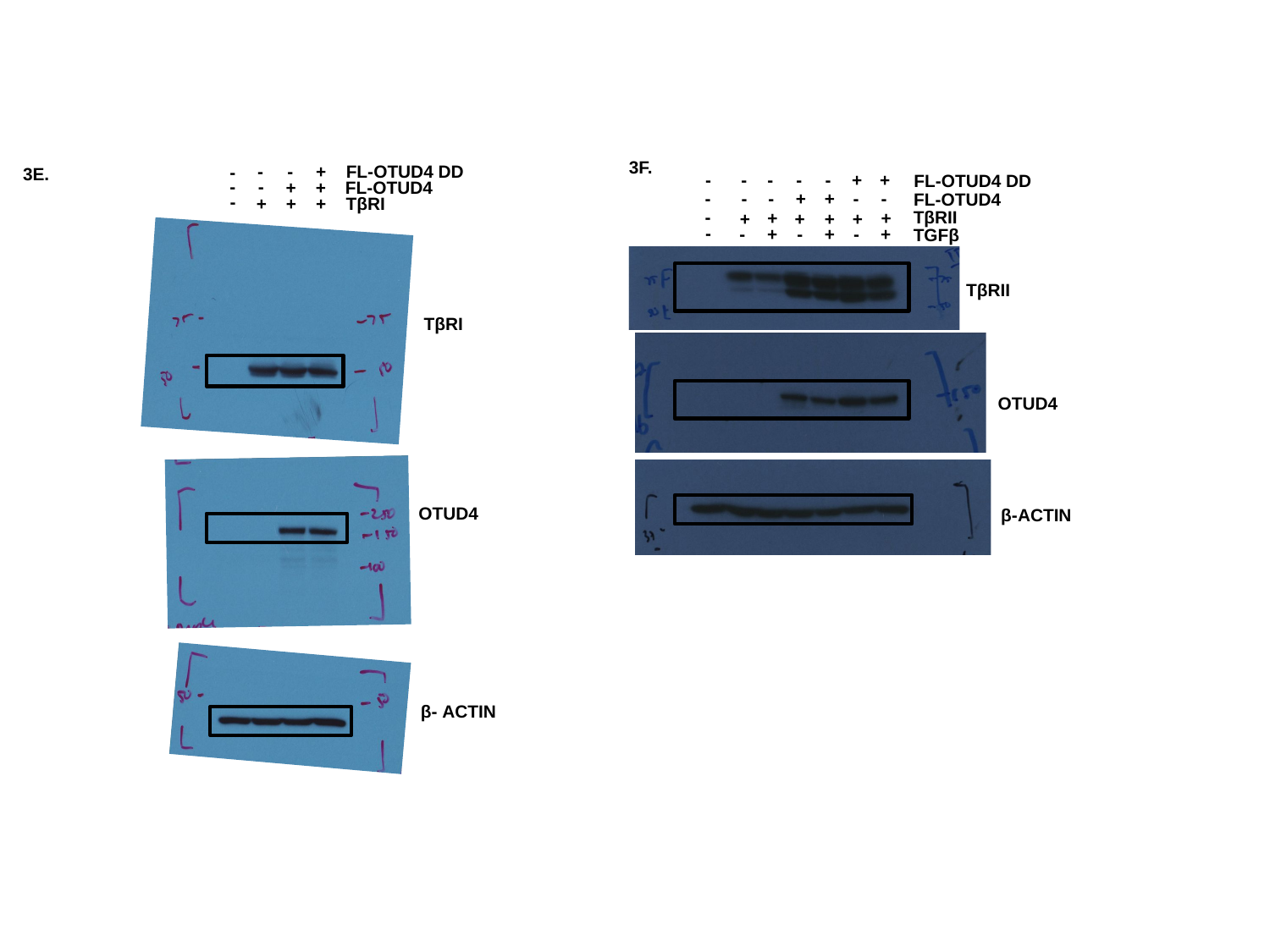

3F.
FL-OTUD4 DD
-
-
+
-
3E.
-
-
-
-
-
+
+
FL-OTUD4 DD
-
-
FL-OTUD4
+
+
-
-
-
+
+
-
-
FL-OTUD4
-
TβRI
+
+
+
-
TβRII
+
+
+
+
+
+
-
-
+
-
+
-
+
TGFβ
TβRII
TβRI
OTUD4
OTUD4
β-ACTIN
β- ACTIN

## Slide 12
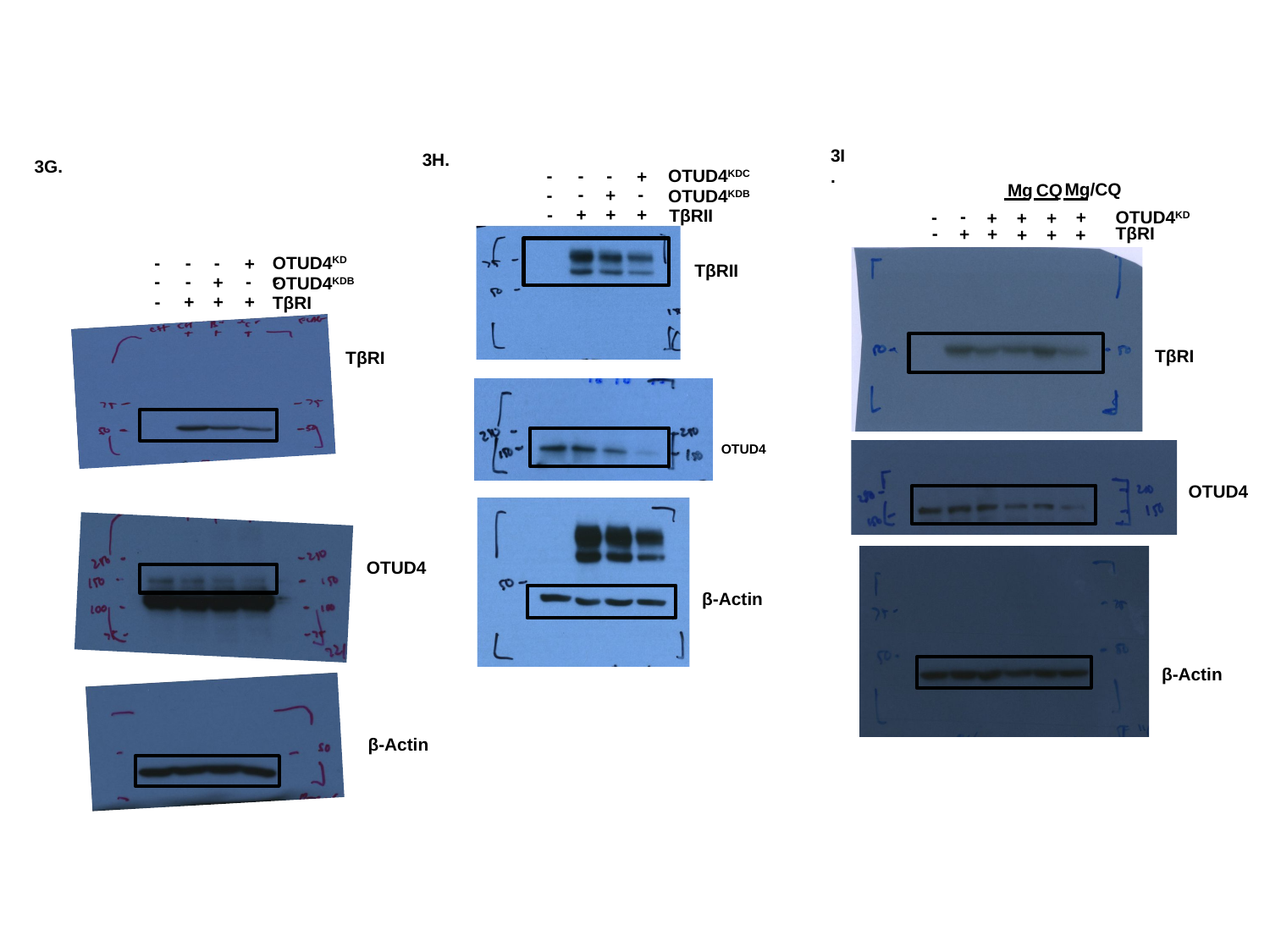

3I.
3H.
3G.
-
-
-
OTUD4KDC
+
-
-
-
+
OTUD4KDB
+
+
+
-
TβRII
Mg/CQ
CQ
Mg
-
OTUD4KD
-
+
+
+
+
-
TβRI
+
+
+
+
+
-
-
-
OTUD4KDC
+
TβRII
-
-
-
+
OTUD4KDB
-
+
+
+
TβRI
TβRI
TβRI
OTUD4
OTUD4
OTUD4
β-Actin
β-Actin
β-Actin

## Slide 13
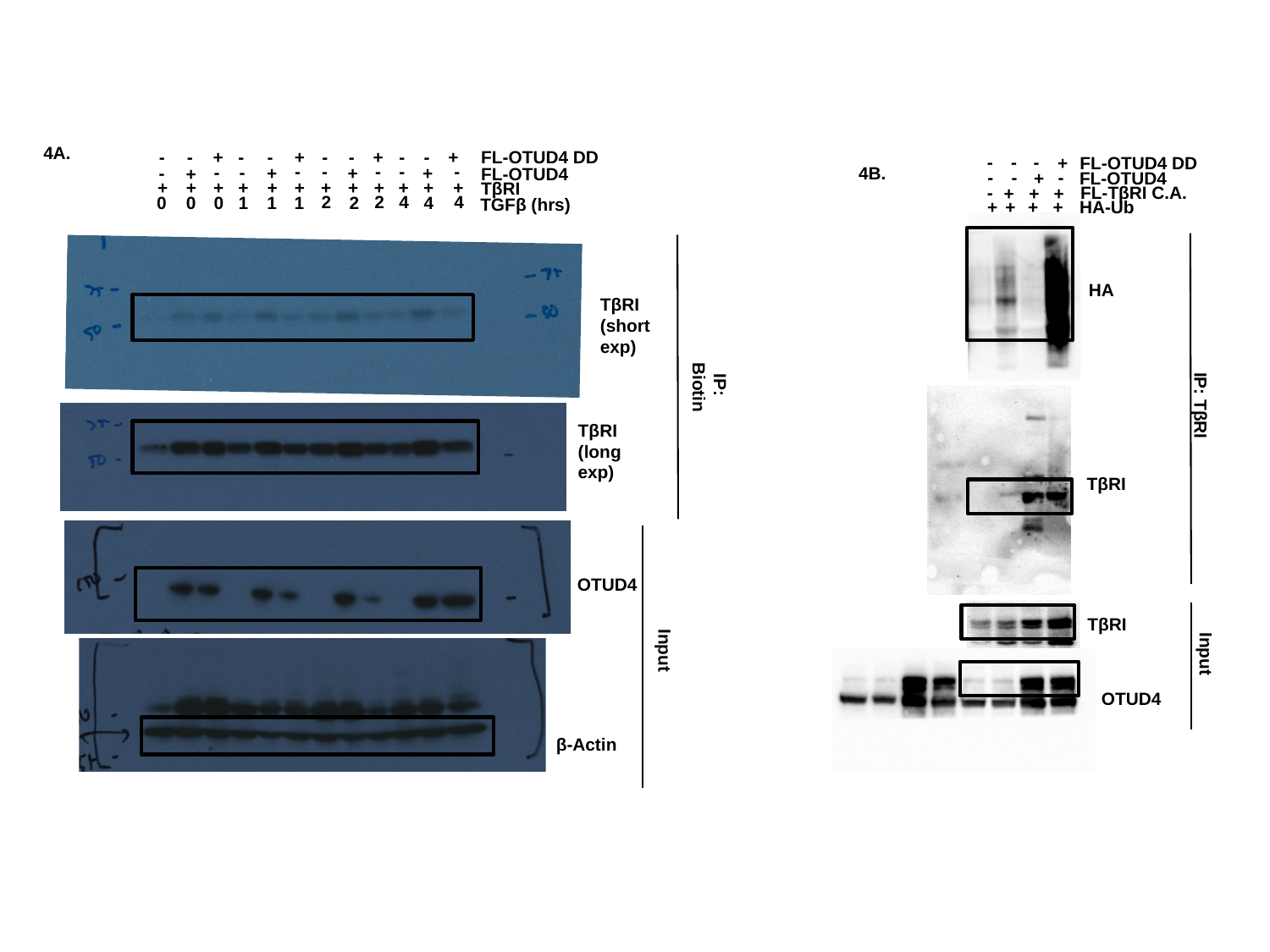

4A.
FL-OTUD4 DD
-
-
+
-
-
+
-
-
+
-
-
+
-
-
-
FL-OTUD4 DD
+
-
-
-
-
-
-
-
-
4B.
+
+
+
FL-OTUD4
+
-
-
-
FL-OTUD4
+
+
+
+
+
+
+
+
+
+
+
+
+
TβRI
FL-TβRI C.A.
-
+
+
+
2
4
4
2
4
1
1
2
1
0
0
0
TGFβ (hrs)
+
+
+
+
HA-Ub
HA
TβRI
(short exp)
IP:
 Biotin
IP: TβRI
TβRI
(long exp)
TβRI
OTUD4
TβRI
Input
Input
OTUD4
β-Actin

## Slide 14
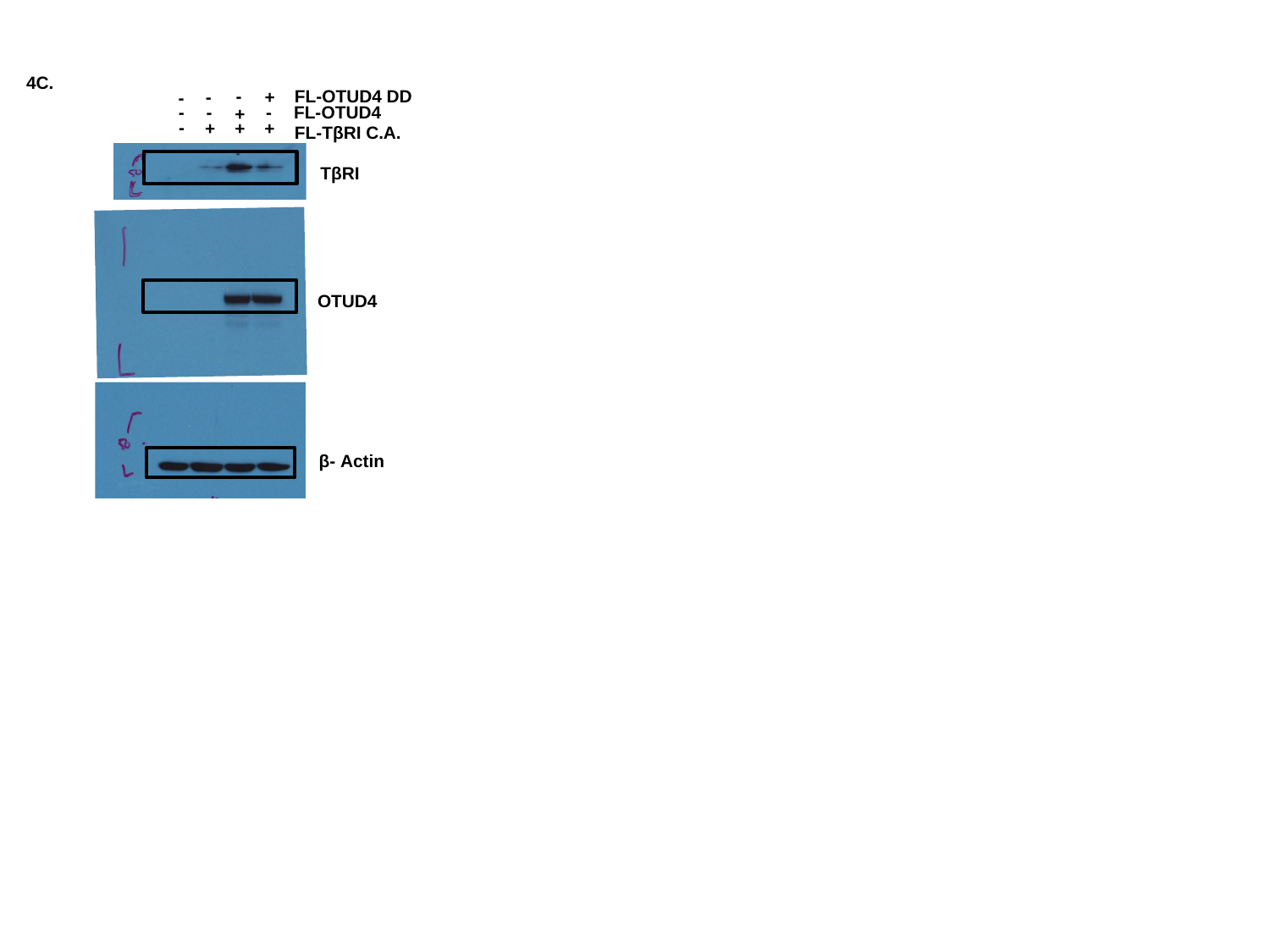

4C.
FL-OTUD4 DD
-
-
+
-
-
-
FL-OTUD4
-
+
-
+
+
+
FL-TβRI C.A.
TβRI
OTUD4
β- Actin

## Slide 15
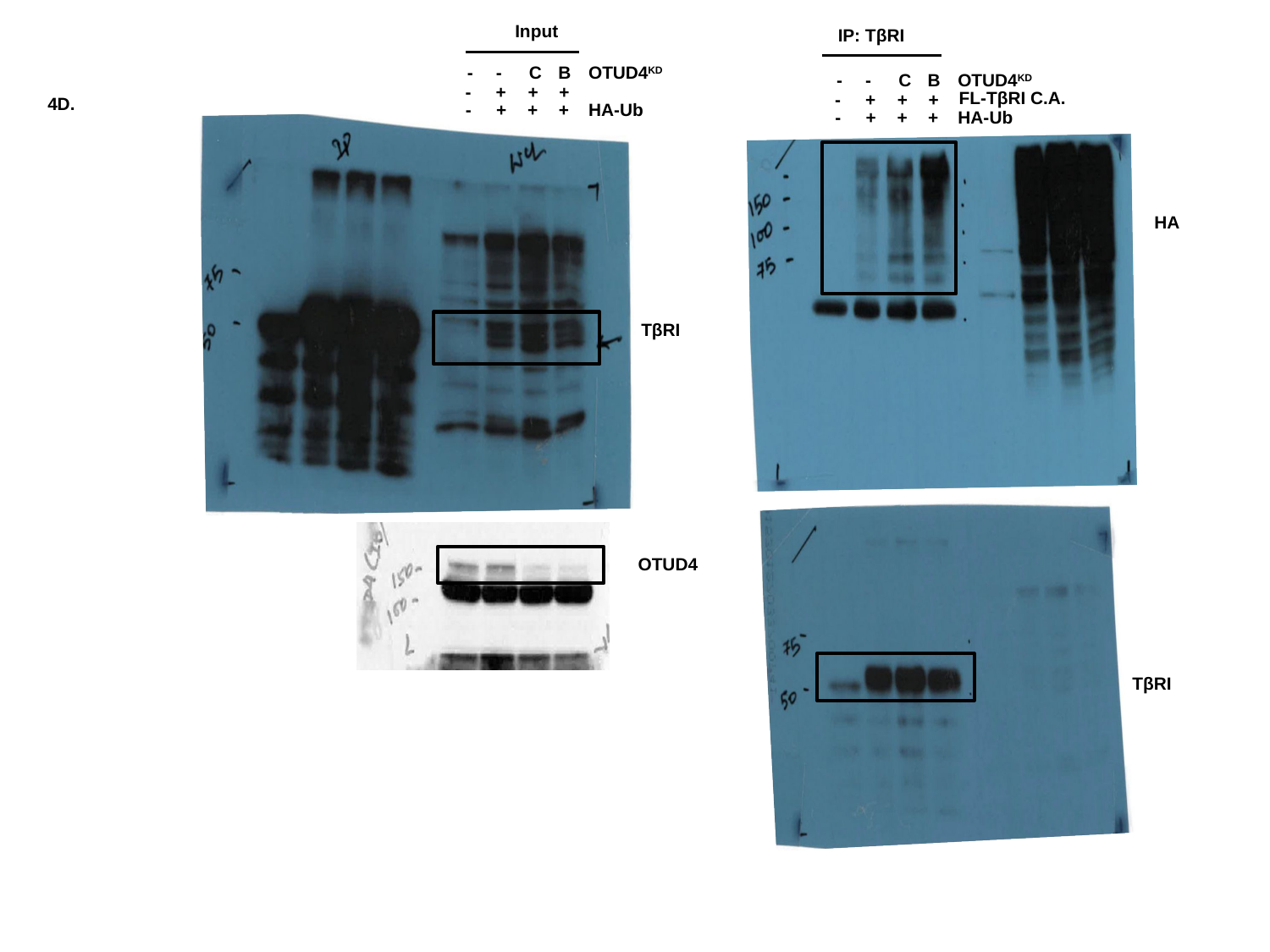

Input
IP: TβRI
-
-
C
B
OTUD4KD
-
-
C
B
OTUD4KD
-
+
+
+
FL-TβRI C.A.
-
+
+
+
4D.
-
+
+
+
HA-Ub
-
+
+
+
HA-Ub
HA
TβRI
OTUD4
TβRI

## Slide 16
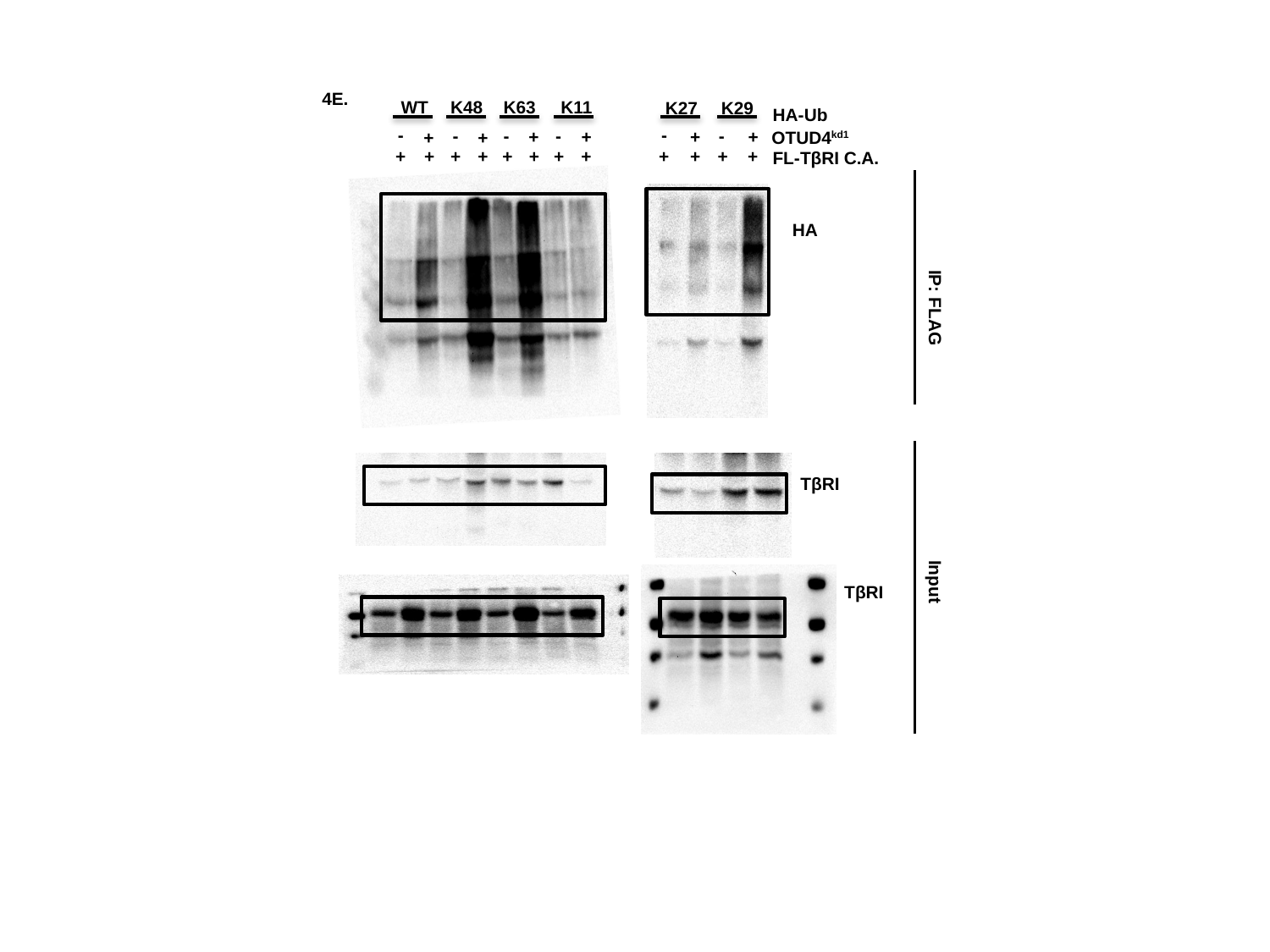

4E.
WT
K48
K63
K11
K27
K29
HA-Ub
-
-
-
-
-
-
+
+
+
+
+
OTUD4kd1
+
+
+
+
+
+
+
+
+
+
+
+
+
FL-TβRI C.A.
HA
IP: FLAG
TβRI
Input
TβRI

## Slide 17
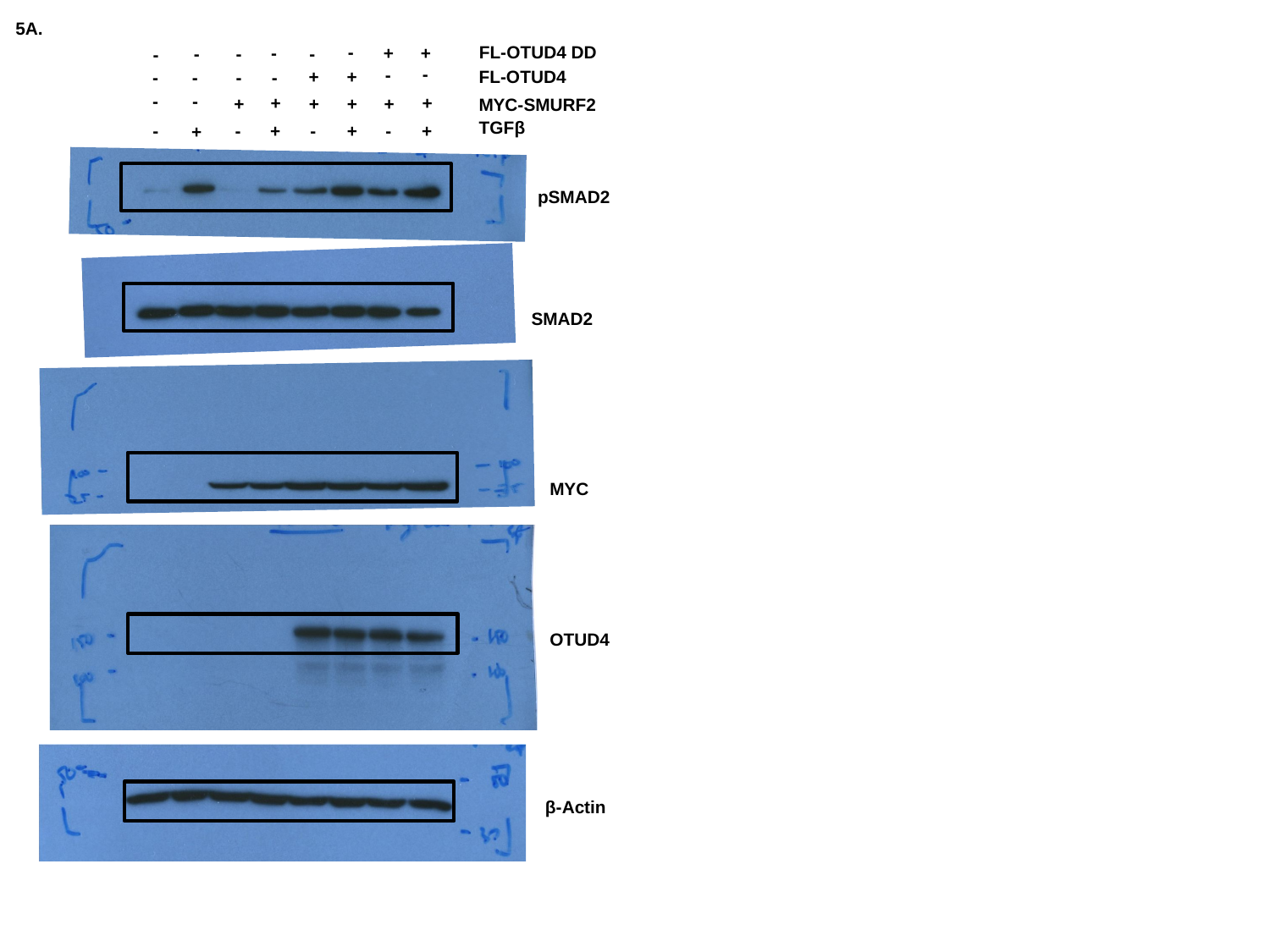

5A.
-
FL-OTUD4 DD
+
-
+
-
-
-
-
-
-
+
+
FL-OTUD4
-
-
-
-
-
-
+
+
+
+
+
+
MYC-SMURF2
TGFβ
-
-
+
-
+
-
+
+
pSMAD2
SMAD2
MYC
OTUD4
β-Actin

## Slide 18
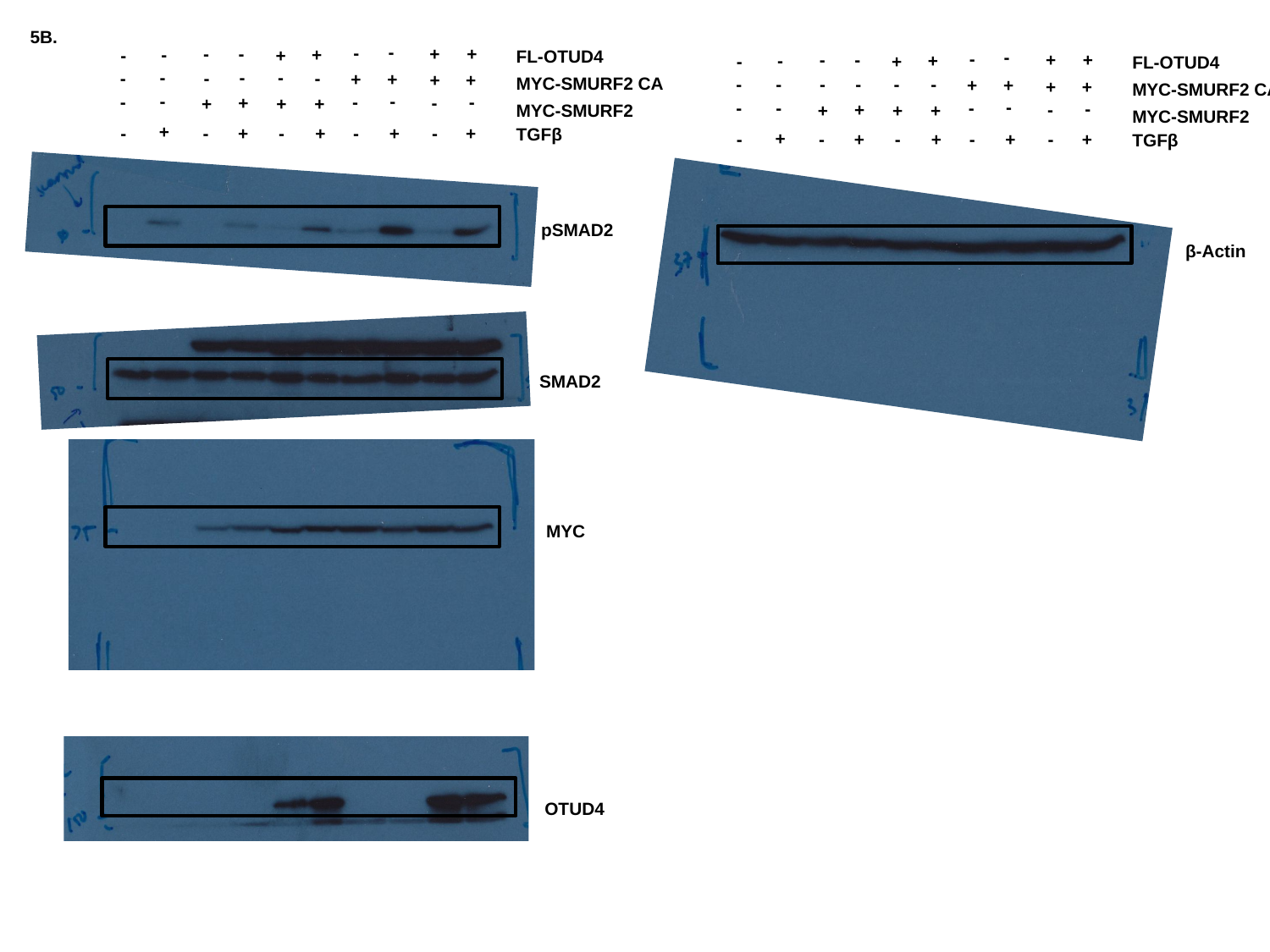

5B.
-
-
-
+
+
-
-
+
+
-
FL-OTUD4
-
-
-
+
+
-
-
+
+
-
FL-OTUD4
-
-
-
-
-
-
+
+
+
+
MYC-SMURF2 CA
-
-
-
-
-
-
+
+
+
+
MYC-SMURF2 CA
-
-
-
-
-
+
-
+
+
+
-
-
-
-
-
+
-
MYC-SMURF2
+
+
+
MYC-SMURF2
+
-
-
+
-
+
-
+
-
+
TGFβ
+
-
-
+
-
+
-
+
-
+
TGFβ
pSMAD2
β-Actin
SMAD2
MYC
OTUD4

## Slide 19
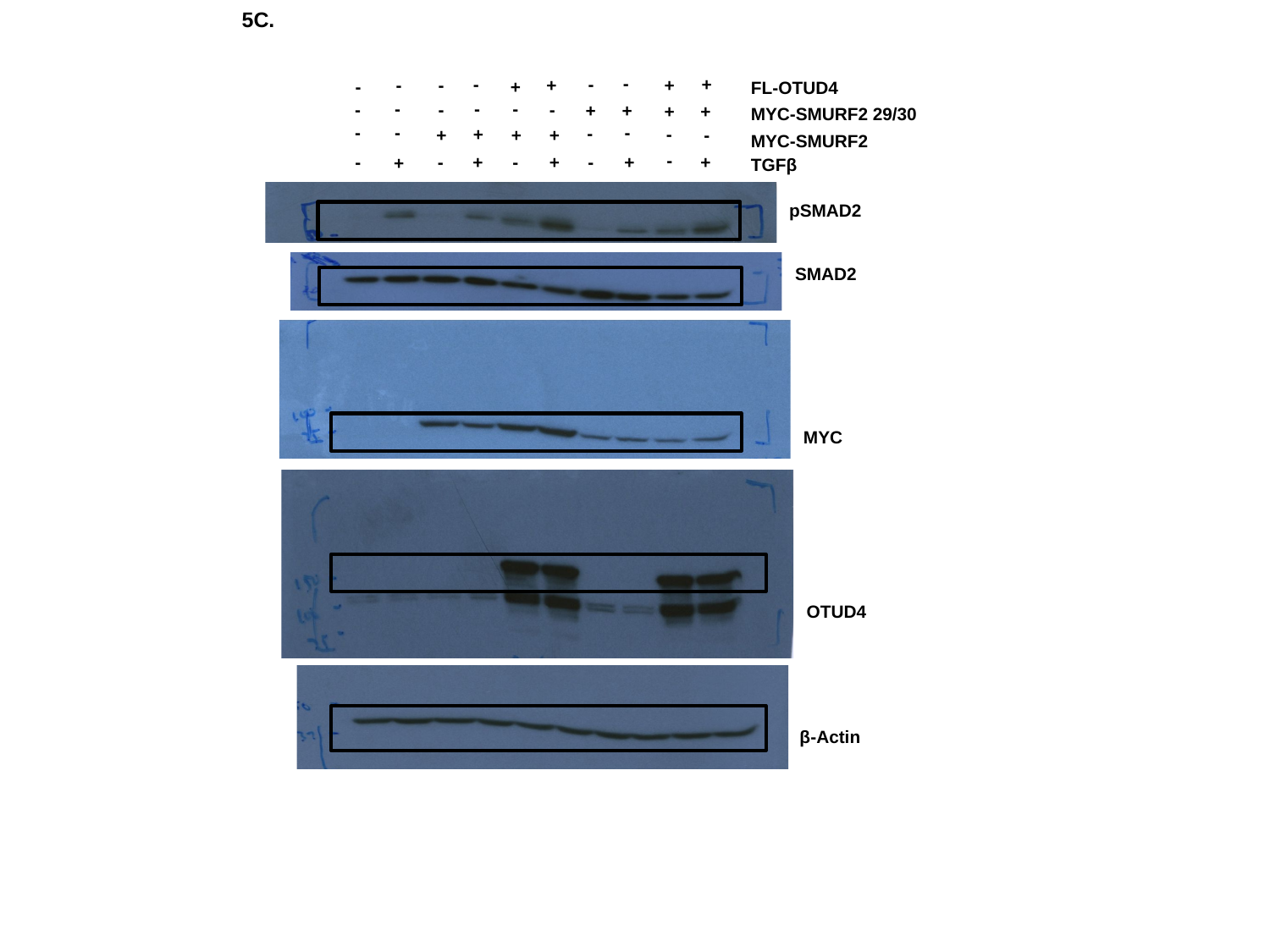

5C.
-
-
-
+
+
-
-
+
+
-
FL-OTUD4
-
-
-
-
-
-
+
+
+
+
MYC-SMURF2 29/30
-
-
-
-
+
-
+
+
+
-
MYC-SMURF2
-
+
-
-
+
-
+
-
+
+
TGFβ
pSMAD2
SMAD2
MYC
OTUD4
β-Actin

## Slide 20
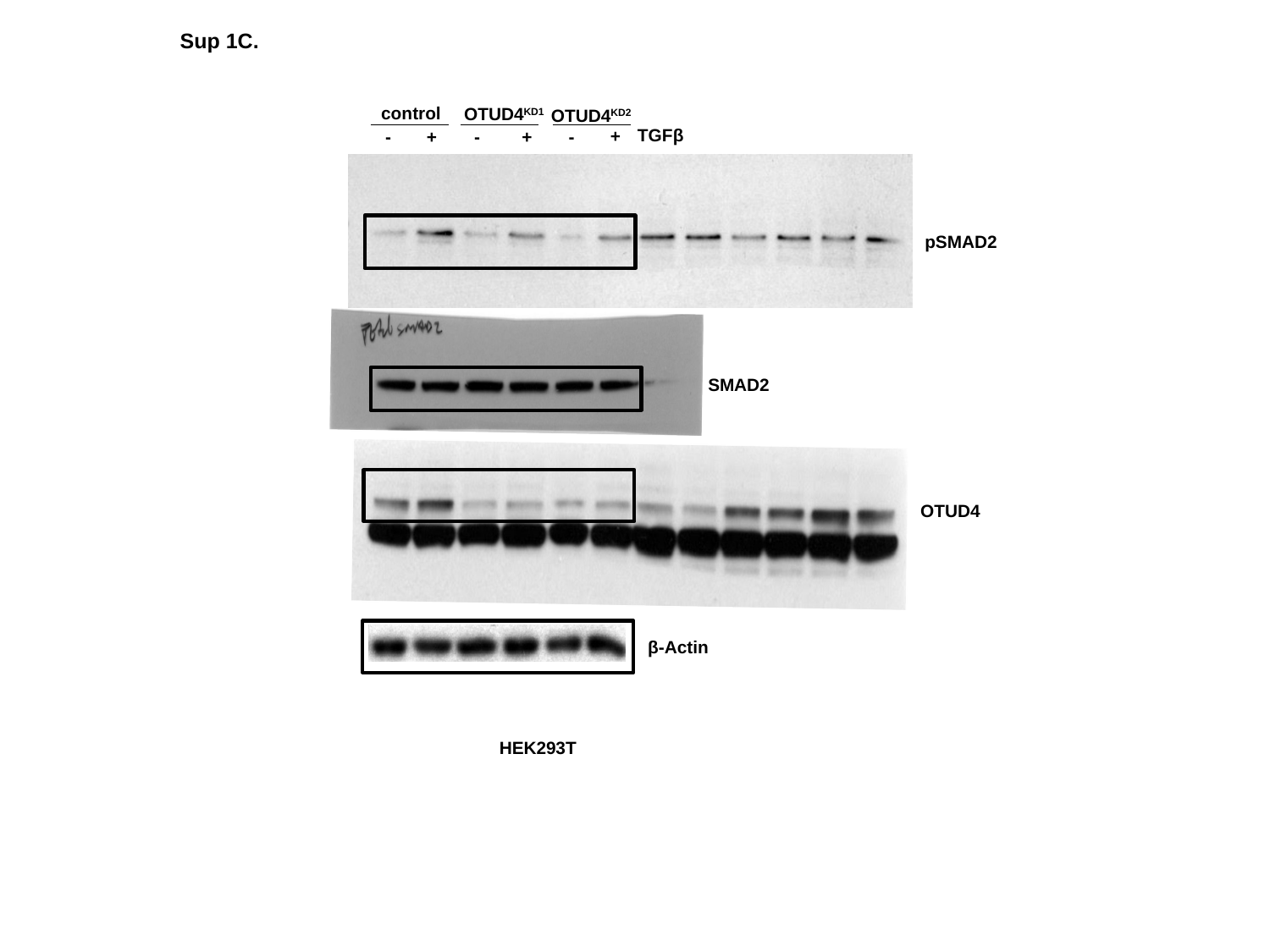

Sup 1C.
control
OTUD4KD1
OTUD4KD2
TGFβ
+
-
-
+
+
-
pSMAD2
SMAD2
OTUD4
β-Actin
HEK293T

## Slide 21
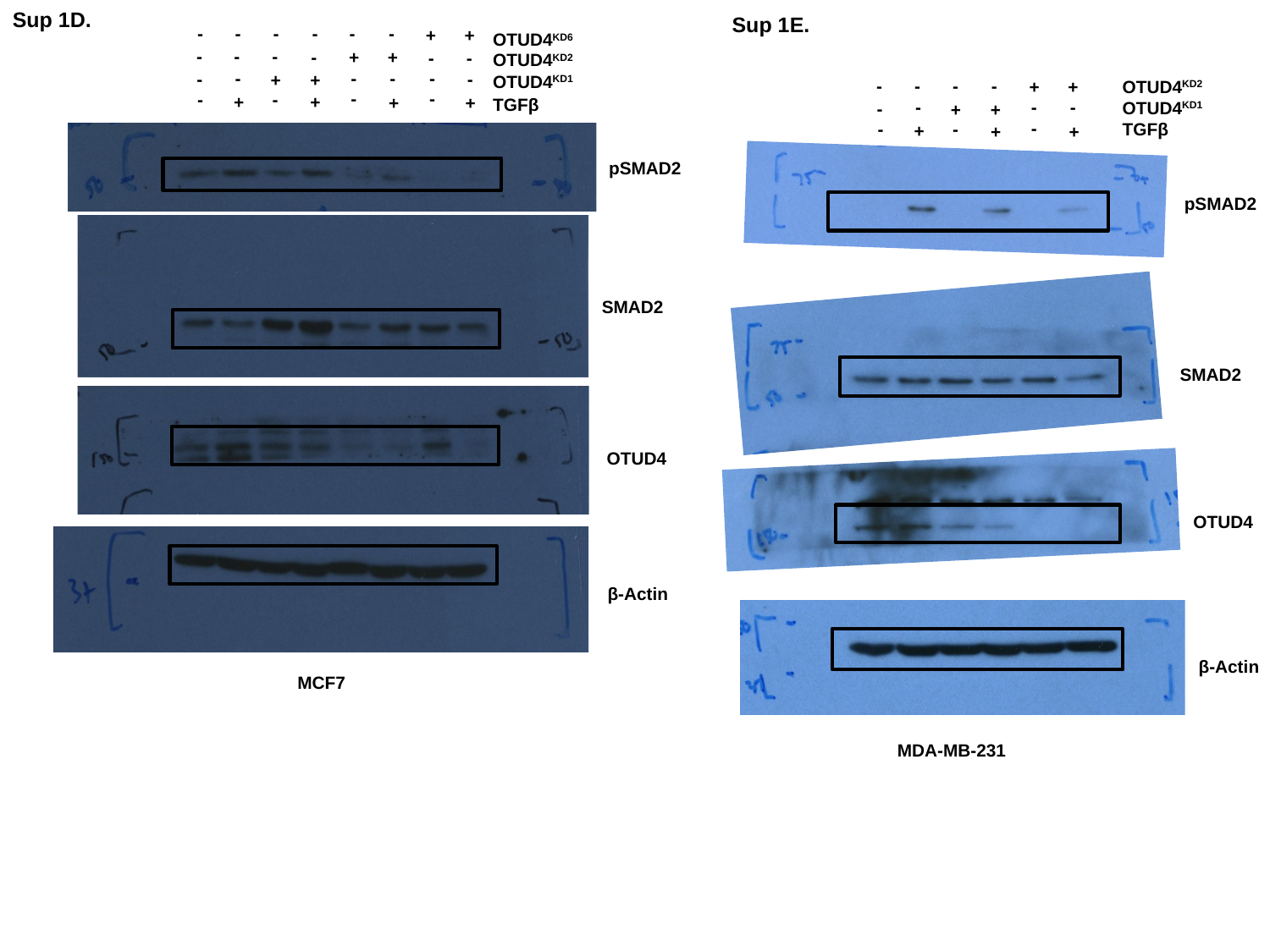

Sup 1D.
Sup 1E.
-
-
-
-
-
-
+
+
OTUD4KD6
-
-
-
-
+
+
-
-
OTUD4KD2
-
-
-
-
-
-
+
+
OTUD4KD1
-
-
-
-
OTUD4KD2
+
+
-
-
-
-
+
+
+
+
TGFβ
-
-
-
OTUD4KD1
-
+
+
-
-
TGFβ
-
+
+
+
pSMAD2
pSMAD2
SMAD2
SMAD2
OTUD4
OTUD4
β-Actin
β-Actin
MCF7
MDA-MB-231
